# Conformational and functional characterization of artificially conjugated non-canonical ubiquitin dimers

**DOI:** 10.1038/s41598-019-56458-z

**Published:** 2019-12-27

**Authors:** Tobias Schneider, Andrej Berg, Zeynel Ulusoy, Martin Gamerdinger, Christine Peter, Michael Kovermann

**Affiliations:** 1 Department of Chemistry, Universitätsstrasse 10, Universität Konstanz, DE-78457 Konstanz, Germany; 2 Department of Biology, Universitätsstrasse 10, Universität Konstanz, DE-78457 Konstanz, Germany; 3 Graduate School Chemical Biology KoRS-CB, Universitätsstrasse 10, Universität Konstanz, DE-78457 Konstanz, Germany; 4 Zukunftskolleg, Universitätsstrasse 10, Universität Konstanz, DE-78457 Konstanz, Germany

**Keywords:** Biophysical chemistry, Computational biophysics, Solution-state NMR

## Abstract

Ubiquitylation is an eminent posttranslational modification referring to the covalent attachment of single ubiquitin molecules or polyubiquitin chains to a target protein dictating the fate of such labeled polypeptide chains. Here, we have biochemically produced artificially Lys11-, and Lys27-, and Lys63-linked ubiquitin dimers based on click-chemistry generating milligram quantities in high purity. We show that the artificial linkage used for the conjugation of two ubiquitin moieties represents a fully reliable surrogate of the natural isopeptide bond by acquiring highly resolved nuclear magnetic resonance (NMR) spectroscopic data including ligand binding studies. Extensive coarse grained and atomistic molecular dynamics (MD) simulations allow to extract structures representing the ensemble of domain-domain conformations used to verify the experimental data. Advantageously, this methodology does not require individual isotopic labeling of both ubiquitin moieties as NMR data have been acquired on the isotopically labeled proximal moiety and complementary MD simulations have been used to fully interpret the experimental data in terms of domain-domain conformation. This combined approach intertwining NMR spectroscopy with MD simulations makes it possible to describe the conformational space non-canonically Lys11-, and Lys27-linked ubiquitin dimers occupy in a solution averaged ensemble by taking atomically resolved information representing all residues in ubiquitin dimers into account.

## Introduction

In eukaryotic organisms essential cellular processes are regulated by the posttranslational modification of proteins using the 76 amino acid comprising polypeptide ubiquitin (Ub)^1^. A set of E1 activating, E2 conjugating and E3 ligating enzymes catalyzes the attachment of one Ub molecule to mainly lysine residues on the target proteins in an ATP-dependent manner. This also occurs on Ub itself, either at one out of its seven intrinsic lysine residues (Lys6, Lys11, Lys27, Lys29, Lys33, Lys48, and Lys63) or at the N-terminal methionine. Usually, an isopeptide bond is formed between the C-terminal carboxyl group of one Ub unit (the distal moiety) and the ε-amino group of a lysine on another Ub unit (the proximal moiety). The resulting Ub dimer (Ub_2_) exhibits distinct topologies and can be optionally expanded by using further conjugations. Various combinations of Ub building blocks are possible and generate unique conformational ensembles depending on the type of linkage used^2,3^. This illustrates the origin of the remarkable functional diversity associated with Ub since surface areas relevant for binding like the hydrophobic patch close to Leu8, Ile44, and Val70^4^ can be presented using differing orientations and distances^3^.

Dimers of the Lys48-linkage type which is well-known for labeling proteins for subsequent degradation by the 26S proteasome^5^ are in a two-state equilibrium between a compact closed and a compact open conformation with an interconversion time of 9 ± 1 ns at nearly physiological pH^6^. In this scenario, the hydrophobic patches of both Ub units either form a contact interface or are solvent exposed and consequently accessible for ligand binding^6,7^. In contrast, Lys63-linked Ub chains which are rather involved in non-degradative processes, e.g. NF-κB activation^8^, intracellular trafficking^9^ or DNA damage response^10^, apparently adopt unconstrained extended structures accompanied by high conformational flexibility^11,12^. Both linkages are known as canonical linkage types as they are extensively explored regarding their structural properties and biological roles^13^. In this study, we focus on two Ub_2_s of non-canonical linkage types, namely Lys11 and Lys27, which are much less understood. A comparison of the free energy landscapes of all Ub_2_s based on coarse-grained and atomistic molecular dynamics (MD) simulations could recently indicate that the Lys11-, and Lys27-linked Ub_2_s exhibit the highest degree of dissimilarity in the conformational space among all linkage types^14^. Here, high-resolution data from NMR spectroscopy were used to experimentally confirm this finding and to better understand the origin of the observed conformational heterogeneity on the molecular level. We outline an approach for combining data obtained by high-resolution NMR spectroscopy and coarse grained and atomistic molecular dynamics (MD) simulations to unravel the conformational states Ub_2_s adopt in a solution averaged ensemble without the necessity to isotopically label both Ub moieties individually.

From a functional perspective, Lys11-linked Ub chains participate in various cellular processes including cytokine signaling^15^, hypoxia response^16^, endocytosis^17^ and endoplasmic reticulum-associated degradation (ERAD)^18^. Of special interest is their role in cell cycle regulation as Lys11-linked Ub chains are highly upregulated during anaphase^19^. At this stage regulator proteins are decorated with Lys11-linked chains by the E3 ligase anaphase promoting complex (APC/C) and are subsequently destructed by the proteasome to terminate mitosis^20,21^. Diverse functions are also reported for Lys27-linked Ub chains, e.g. in the processes of mitophagy^22^, DNA repair^23^, antiviral immunity response^24^ and neuronal protection in Parkinson’s disease^25^. In addition, both linkage types are implicated to have regulatory effects on the Ub code as the Lys11-linkage enhances the signal for proteasomal protein degradation in mixed Lys11/Lys48-linked Ub chains^26^ and Lys27-linkage prevents other Ub chain types from cleavage by deubiquitinases (DUBs)^27^.

Two structures based on crystallographic data of Lys11-linked Ub_2_s have been published so far which structurally differ in terms of domain-domain orientation^19,28^. An NMR-derived three-dimensional solution averaged structure of the Lys11-linked Ub_2_ fails to cover these two structures in the conformational space of the ensemble in solution^29^. In contrast, the crystallographic structure obtained for a Lys27-linked Ub_2_ corresponds to a high degree to the NMR-derived structure. This potentially indicates reduced inherent dynamics present in this type of linked Ub_2_ compared to Lys11-linked Ub_2_ as the flexibility of the isopeptide linker is spatially constrained due to its buried orientation in the proximal moiety^27,30^.

In the present study, we generated Lys11- and Lys27-linked Ub_2_s in a semisynthetic approach. Both moieties were expressed recombinantly in *E. coli* cells and have been subsequently conjugated using biorthogonal click chemistry^31,32^. This methodology results in a triazole-linkage between the proximal and the distal moieties which is comparable in terms of length and electronic properties to the native isopeptide bond (Fig. S1)^33^ and, as an advantage, it cannot be cleaved by DUBs^34^.

The biological functionality of Ub chains produced in this way has been already successfully demonstrated in an affinity enrichment assay^32^. Along these lines, it has recently been shown that Ub_2_ based on triazole linkage at the canonic position Lys48 mirrors structural and dynamical features seen for isopeptide Lys48-linked Ub_2_ very reliably. Using this approach, we are capable to implement segmental isotopic labeling of the proximal Ub moiety within the dimer^35^. Consequently, these species are well suited for high-resolution NMR studies because they avoid potential signal overlap of corresponding resonances originating from both Ub_2_ units. Hence the structural and dynamic impact which the distal moiety on the proximal site has been precisely probed at a residue-by-residue basis. This has been performed here for the two non-canonically Lys11-, and Lys27-linked Ub_2_s and – for comparison – for the canonically Lys63-linked Ub_2_. In combination with structures of the respective isopeptide-linked Ub_2_s which were obtained from extensive conformational ensembles produced by MD simulations, we propose a model at atomic resolution for the domain-domain orientation between the two moieties of the respective dimers in a solution averaged ensemble. Advantageously, intertwining NMR spectroscopy with MD simulations in this manner avoids additional isotopic labeling and subsequent acquisition of high-resolution NMR data of the residues comprising the distal moiety.

As Ub’s and Ub chains’ functionality is highly dependent on its inherent dynamic characteristics^36^, we additionally probed intrinsic dynamics on different time scales. Internal motions on a fast picosecond to nanosecond time scale comparing Lys11-, and Lys27-linked Ub_2_s were probed by NMR spin relaxation measurements and complemented with root mean square fluctuations (*RMSF*) obtained by MD simulations. Information about potential domain-domain motions of Ub_2_ on a slower millisecond time scale has been revealed by amide proton exchange NMR measurements whereas diffusion NMR methodology has been applied to monitor the hydrodynamic dimensions of Ub_2_s. Importantly, the specific structural response of Lys11-, Lys27-, or Lys63-linked Ub_2_s when recognizing an Ub binding domain illustrates the functionality of the Ub_2_s which have been assembled by the artificial linkage presented here. Based on our data we propose that the balanced conformational flexibility seen for the two individual units present in Ub_2_ plays a major role for the functional variety of polyubiquitin chains^37^.

## Results and Discussion

### Cysteine mutation and adding an artificial linker on monomeric Ub

**Figure 1:**
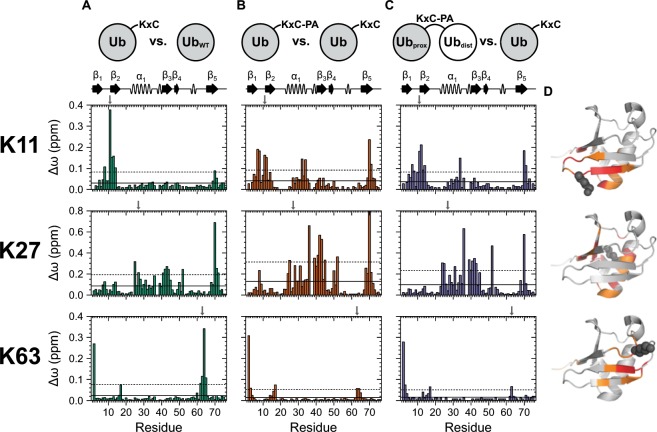
Chemical shift perturbation (CSP, Δω) mapping of Ub variants. Comparisons are shown for: (**A**) monomeric cysteine mutants UbK11 C, UbK27C, and UbK63C versus monomeric wild type Ub, (**B**) monomeric cysteine mutants possessing propargyl acrylate (PA) linker UbK11C-PA, UbK27C-PA, and UbK63C-PA versus corresponding cysteine mutants lacking PA linker and (**C**) proximal moieties of artificially Lys11-, Lys27-, and Lys63-linked Ub_2_s versus corresponding monomeric cysteine mutants (note the different scaling for Lys27 compared to Lys11 and Lys63). The horizontal lines indicate Δω values larger than the mean (continuous mode) and larger than the mean plus one standard deviation (dotted mode). Secondary structural elements according to PDB ID 1D3Z are indicated on top and the site used for conjugation has been highlighted by using a vertical arrow. (**D**) The same structure has been used to highlight residues possessing CSP values larger than the mean (colored in orange) and larger than the mean plus one standard deviation (colored in red) based on data shown in C. Side chain atoms of the lysine residue used for cysteine mutation, PA attachment and the conjugation of the distal moiety are shown as spheres and are colored in dark gray. The structures have been created by using the PyMOL Molecular Graphics System, Version 2.4.0a0, Schrödinger, LCC (www.pymol.org).

The location of the lysine residue in the proximal Ub unit which is used for linkage defines per se the relative position of the distal unit within Ub_2_, thus inherently constraining the conformational space compared to two Ub proteins which are not covalently bound^38,39^. Considering this property as the most specific determinant for the characterization of linkage-dependent structural ensembles of Ub_2_s, we first investigated the effect of a single lysine-to-cysteine mutation and the adding of an artificial linker on monomeric Ub which are prerequisites for the subsequent formation of Ub_2_s. Thus, we acquired two-dimensional heteronuclear ^1^H-^15^N HSQC NMR spectra of monomeric UbK11C, UbK27C, and UbK63C as well as hydrophobic propargyl acrylate (PA) containing species and compared the corresponding chemical shifts of the backbone amide proton and nitrogen resonances with those representing monomeric wild type Ub (Fig. 1A). On the basis of the strong signal dispersion with a pattern typical for Ub as seen in all spectra (Figs. S2A–D, S3A–D, and S4A–D), we conclude that the tertiary Ub fold is conserved in all mutants used in this study which is in agreement with respective MD simulations (Fig. S5). However, calculation of chemical shift perturbations (CSPs) revealed differences indicating local structural rearrangements which are specific for the mutation site used. Since the substitution of one residue changes the chemical environment in its closer proximity, inevitably the highest CSP values are expected to be next to the mutation site in all species. Indeed, this is the case for UbK11C and UbK63C but does not hold for UbK27C which exhibits a more complex picture (Fig. 1A).

First, we present the structural alterations that are induced by mutation of residue Lys11 to cysteine. With regard to the NMR solution structure of monomeric wild type Ub (PDB ID 1D3Z), Lys11 is localized in the β_1_/β_2_-loop and its side chain points to the C-terminal end of the central α-helix where it forms a salt bridge with the side chain carboxyl group of Glu34^40,41^. As a cysteine residue lacks a positive charge and differs in length compared to lysine, this salt bridge has to be interrupted in UbK11C (Fig. S5A). Structural information obtained from MD simulations indicates that this salt bridge is replaced by a hydrogen bond between Cys11 and Glu34 which leads to a significant structural change in the region between Leu8 and Ile13 (Fig. S5A). However, perturbations near Glu34 in the C-terminal part of the helix are rather transient (Fig. 1A). It is reasonable that the corresponding amide proton and nitrogen resonances are not sensitive in this case, because their environment is primarily defined by residues forming a helix shielded by their side chains. CSPs in this region (Lys27, Lys29-Gly35) become more pronounced only when the attachment of the space-consuming PA linker amplifies the disturbance of the structure (Fig. 1B). The strongest perturbations are experienced by Cys11 itself and residues next in the sequence including the loop region (Thr7-Gly10) and the N-terminal end of the β_2_-strand (Thr12-Thr14) (Fig. 1B). In consequence, changes on Val70 can be explained by the impact on Leu8, because both residues are part of the dynamic hydrophobic surface patch (Fig. S5A). A modification in the β_1_/β_2_-loop thus influences the conformational equilibrium of this dynamic feature, an effect that is also increased by the attachment of PA.

The second cysteine mutation in the present study concerns Lys27 which is located in the center of the α-helix, with its side chain protruding into the hydrophobic core of Ub. Thus, a multitude of residues compassing round the molecular center may consequently recognize a substitution at that position. Therefore, large CSP values are obtained for residues in the center of the α-helix (Asn25, Val26, Lys27, Ala28, Leu30, Gln31) and the opposing β_3_- (Gln41-Phe45) and β_5_-strands including a part of the C-terminal tail (His68-Arg72) (Fig. 1A). As some of those secondary structural elements harbor residues contributing to the hydrophobic surface patch, structural changes induced in this area slightly extend to Leu8 in the β_1_/β_2_-loop (Fig. 1A). Strong perturbations on Ile36 and Asp39 also implicate an impact on the first 3_10_-helix and the preceding unstructured region (Fig. 1A), although the appendant proline residues in between (Pro37, Pro38) are not detectable in this NMR experiment. In addition, a salt bridge between the side chains of Lys27 and Asp52 has to be disrupted in case of a cysteine mutation (Fig. S5B)^42^. This is in agreement with CSP values observed for Asp52 itself and adjacent Leu50 (Fig. 1A) as well as structural rearrangements found in accompanying MD simulations (Fig. S5B). Similar to UbK11C the salt bridge (between Lys27 and Asp52) is substituted by a hydrogen bond, in this case between Cys27 and Asp52. Since this contact is much shorter compared to the salt bridge the α-helix is tilted to some extent (Fig. S5B). Notably, another cluster of perturbations is found in the β_2_-strand (Ile13, Thr14) which is far away from the mutation site (Fig. 1A). This might be associated with local rearrangements in the core, affecting hydrophobic interactions with Ile13^43,44^. Generally, the same regions which are perturbed by the cysteine mutation are also impacted by PA linker attachment but to a greater extent (Fig. 1B). This suggests that the origin of the CSPs is principally based on different steric requirements of lysine and cysteine side chains and the PA linker, respectively. The hydrophobic character of PA may additionally contribute to those changes.

Only few residues in Ub show significant CSP values in consequence of the cysteine mutation on Lys63. Most residues which are affected here refer to the loop region between the second 3_10_-helix and the β_5_-strand (Gln62-Ser65) where Lys63 is located (Fig. 1A). Interestingly, largest perturbations are obtained for Glu64 next to the mutation site and Gln2 in the neighboring β_1_-strand (Fig. 1A). Both are connected via a hydrogen bond between the amide proton of Glu64 and the carbonyl oxygen of Gln2^45^. The MD simulations are capable to explain these perturbations by an interaction between the thiol group of the Cys63 side chain with the amide proton of Gln2 (Fig. S5C). This results in a local structural disruption at the N-terminus that is also sensed by Val17 which is located in the adjacent β_2_-strand and connected with Met1 via another backbone hydrogen bond (Fig. 1A)^45^. Remarkably, reaction of the thiol group with the PA linker neither recovers the chemical shifts of Glu64 nor of Val17 in comparison to wild type Ub (Fig. 1B). PA enhances the changes of their chemical environments significantly, similar to both of the other non-canonical cysteine mutations.

For all cysteine mutants probed here we note that the level of perturbations is basically depending on the density of residues in spatial proximity to the mutated amino acid. For example, the side chain of Lys27 is completely buried inside the molecule and surrounded by multiple side chains that are able to sense this mutation whereas Lys63 is exposed to the solvent and does not interact with many other residues. This inversely correlates with the solvent accessible surface area (SASA) of the mutated residues. A decreasing number of significant CSP values can thus be observed from UbK27C to UbK11C to UbK63C (Fig. 1A), whilst SASA values increase from Lys27 to Lys11 to Lys63 correspondingly^46^.

### Structural impact of adding distal Ub on the proximal moiety

Apart from local structural rearrangements described above, the integrity of the tertiary fold of monomeric Ub could be confirmed for all cysteine mutated Ubs under study. Thus, they have been used subsequently as building blocks representing the proximal entity for the formation of synthetically linked Ub_2_s comprising a heterocyclic triazole ring instead of the native isopeptide bond (Fig. S1). Note that the notation of artificially PA-linked Ub_2_s used in the present study does not explicitly indicate the lysine-to-cysteine mutation. Two-dimensional ^1^H-^15^N HSQC NMR spectra have been acquired illuminating the isotopically labeled proximal moiety in the corresponding dimer. The resulting data have been compared to spectra of monomeric cysteine mutated Ubs that represent the monomeric building blocks of the respective dimers (Fig. 1C). Consequently, the calculated CSP values result from structural alterations in the proximal moiety caused by either the artificial linker or the vicinity to the distal moiety.

The artificially Lys11-linked Ub_2_ exhibits patterns of significant CSP values for residues comprising the β_1_- to the β_2_-strand (Val5-Leu8, Gly10-Leu15), the region at the C-terminal end of the α-helix (Lys27, Lys29, Gln31-Glu34, Ile36) and the spatial proximity of Val70 (Arg42, Val70, Leu71, Leu73) (Fig. 1C,D). CSP values for the artificially Lys27-linked Ub_2_ cluster at residues comprising (i) the α-helix (Glu24-Val26, Ala28, Lys29, Gln31, Lys33-Gly35), (ii) the succeeding loop region including the first 3_10_-helix and the β_3_-strand opposing to the conjugation site (Ile36, Asp39-Leu43, Phe45) and (iii) the C-terminal β_5_-strand (His68, Val70, Leu71) (Fig. 1C,D). Besides, Asp52 is also perturbed to a large extent as explained above by the disruption of a salt bridge (Fig. 1C,D). Note that elements comprising the proximal moiety within Ub_2_ that have already been perturbed by the corresponding cysteine mutation (Fig. 1A) are affected for both non-canonical linkage types (Fig. 1C). Hence, we conclude that those elements are specifically manipulated during dimer formation and are characteristic for the type of linkage. However, when comparing the spectra of Ub_2_s with those of cysteine mutated monomeric Ubs having the PA linker already attached, amplitudes of the CSPs are relatively small (Fig. S6A,B). This indicates that the conformational change the proximal unit experiences due to dimerization is primarily defined by the position of the conjugation site (and the conjugation itself) rather than direct interaction with the distal unit. Considering that the route of the trajectories traced by the cross peaks present in two-dimensional heteronuclear ^1^H-^15^N HSQC spectra is not consistently linear starting from monomeric wild type Ub, to monomeric cysteine mutated Ub lacking or possessing the PA linker and finally to PA-linked Ub_2_ (Fig. S7A–C), this conformational change cannot be described as concerted event. Nevertheless, since almost the same residues are perturbed during all stages of the process of dimer formation (Fig. 1A,B, and S6), the final linkage-specific conformation might be approached stepwise by different events along the conformational path. Potential hydrophobic interactions induced by PA which diminish when the triazole ring is formed may contribute to this route of trajectories. Although the triazole-linked Ub_2_s resemble their native counterparts quite accurate as indicated by the strong similarity to the CSP mappings for the isopeptide-linked Ub_2_s obtained by Castañeda *et al*. (Fig. S8)^27,37^, the conformational pathway of the semisynthetic approach differs from the enzymatic coupling performed by nature.

In case of artificially Lys63-linked Ub_2_ perturbations originate exclusively from the conjugation process (Fig. 1C,D). No significant changes are apparent when comparing the NMR spectra acquired for the dimer and the corresponding monomeric cysteine mutated Ub possessing the PA linker (Fig. S6C). This is in strong agreement with the assumption of an extended structure as proposed for canonically Lys63-linked Ub_2_s and Ub chains conjugated by isopeptide linkage^11,12^. Due to the rather unconstrained flexibility of both units in this arrangement, the distal Ub_2_ moiety is not expected to exert a strong impact on residues comprising the proximal one.

The comparison of CSP values originated from artificial conjugation of Ub_2_s based on triazole linkage with isopeptide conjugation illuminates a strong correlation for the three different linkages probed in the present study (Fig. S8). Remarkably, residues comprising the proximal moiety in Lys11-, and Lys63-linked Ub_2_ show even quantitatively the same structural response when comparing the monomeric proximal moiety with Ub_2_. A sequence dependent analysis of CSP values performed for Lys27-linked Ub_2_ shows that there is also a significant correlation (qualitatively as well as quantitatively (except Val70)) between both values when sequence positions Gln2-Ile23 and Gly47-Gly76 are compared. The analysis of CSP values for positions Glu24-Ala46 shows (except for Ile36) a rather qualitative agreement between both. This behavior is potentially based on Lys27 to Cys27 replacement which causes a tilt of the α-helix comprising residues as suggested by MD simulations (Fig. S5B).

In parallel, residues which are not affected via isopeptide conjugation in Lys11-, Lys27-, and Lys63-linked Ub_2_ are also not affected by utilizing triazole linkage (Fig. S8). This observation holds for Lys27-linkage as well. Consequently, this comparison of CSP values gives strong support for the reliability of the chemical approach applied here for artificial conjugation of Ub_2_s. Moreover, an almost perfect overlay of one-dimensional ^1^H NMR spectra which have been acquired for both Lys11-, and Lys27-linked Ub_2_s before and after long-term storage of more than one year shows the strong inherent resistance of such assembled Ub_2_s against degradation (Fig. S9). Thus, we propose that triazole-linked Ub_2_s can be used as ideal surrogates in biochemical and biophysical studies.

### Conformational equilibrium of Ub dimers

**Figure 2:**
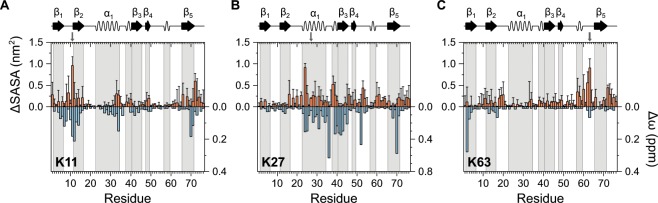
Comparison between chemical shift perturbations obtained by using NMR spectropscopy, Δω (colored in blue, y-axis on the right), and changes in the solvent accessible surface area obtained by using MD simulations, ΔSASA (colored in orange, y-axis on the left). Chemical shift perturbations originating from Fig. 1C are compared to computed ΔSASA values obtained for the proximal unit of natively isopeptide-linked Ub_2_s versus monomeric wild type Ub. The results are shown for (**A**) Lys11-, (**B**) Lys27-, and (**C**) Lys63-linkage type. Secondary structural elements according to PDB ID 1D3Z are indicated on top and highlighted by using a background colored in gray.

As CSP values originated for proximal units in PA-linked Ub_2_s result – at least partially – from domain-domain contacts with their distal units, they inherently contain valuable information about the relative domain-domain orientation between the two moieties in a solution averaged ensemble. Moreover, it has recently been reported for natively isopeptide-linked Ub_2_s that a high correlation exists between experimental CSPs and the simulated residue-wise loss of SASA^14^. This is because regions on a protein which are in contact with a second protein experience a change in SASA which is accompanied by a change of the chemical environment. We calculated residue-wise differences in the SASA (ΔSASA) from simulations started from four (for each linkage type) low-energy conformations of natively isopeptide-linked Ub_2_s by comparison of the proximal unit with simulation of monomeric wild type Ub. The resulting ΔSASA values were then compared to the CSPs obtained for the artificially PA-linked Ub_2_s versus their corresponding monomeric lysine-to-cysteine mutants (Fig. 2). This was performed for the non-canonical Lys11- and Lys27-linkage as well as for the canonical Lys63-linkage type in order to gain coherent information about the domain-domain orientation of different Ub_2_s.

Starting with Lys11-linked Ub_2_ which exhibits a remarkable correlation between ΔSASA and CSP values (Fig. 2A), three regions show pronounced effects for both parameters, (i) near the β_1_/β_2_-loop, (ii) the C-terminal end of the α-helix and (iii) the β_5_-strand including the C-terminal tail (Fig. 2A). Those regions define an area on the molecular surface of the proximal unit in Ub_2_ that is presumably covered by the distal moiety in a solution averaged ensemble. Thus, those regions allow a reconstruction of the favored position of the distal unit (Fig. S10A). It is striking that CSPs are more widespread in the region near the β_1_/β_2_-loop than the ΔSASA values (Fig. 2A). This is because the side chains of succeeding residues in the β_1_- and β_2_-strands are pointing into opposite directions explaining the alternating pattern of ΔSASA values within those elements (Fig. S11A). Consequently, we conclude that this region is underrated when determining the orientation of the distal unit. At the C-terminal end, residues located at the end of the β_5_-strand show CSP values highest in amplitude whereas ΔSASA values exhibit a maximum at the start of the tail region (Fig. 2A). The slight shift of the maxima might be based on the different conditions that the amino acids are subjected to in the β-sheet or at the flexible tail. The chemical environment of a residue present in a β-sheet is inherently more defined than a residue present in a tail region leading to differences in the potential amplitude of CSPs. Contrary, SASA is generally higher in the tail region and can potentially be reduced much more easily in the presence of the distal moiety in Ub_2_ than the SASA for a residue present in a β-sheet. Overall, the qualitative agreement of the CSPs and the simulation-based ΔSASA analysis performed on Lys11-linked Ub_2_ is excellent.

In contrast to the Lys11-linkage, ΔSASA values of the Lys27-linked Ub_2_ do not match the corresponding CSPs completely (Fig. 2B). Significant correlation is found at the central α-helix which harbors the conjugation site (Fig. 2B). In addition, noticeable ΔSASA values are identified for residues located in the first 3_10_-helix (Fig. 2B). Although the two proline residues (Pro37 and Pro38) partly involved cannot be probed by two-dimensional ^1^H-^15^N HSQC NMR spectroscopy, residues of this 3_10_-helix are also implicated to have their chemical environment changed due to the strong perturbation of the adjacent Ile36 (Fig. 2B). Besides, several other clusters of residues are apparent in Lys27-linked Ub_2_ which possess either significant CSP or ΔSASA values (Fig. 2B). Accordingly, high ΔSASA values can be observed in the preceding loop of the α-helix with an alternating pattern as described above for the Lys11-linkage type as well as in the unstructured region between the β_4_- and β_5_-strands except of the second 3_10_-helix in between (Fig. 2B). CSP values, by contrast, are exclusively high in the succeeding loop of the α-helix and the joining β_3_-strand as well as at the C-terminal end of the β_5_-strand (Fig. 2B). Since ΔSASA values are based on simulated data from the natively isopeptide-linked Ub_2_ whereas CSPs are obtained from experimental NMR measurements of the corresponding artificially PA-linked Ub_2_, the discrepancies can be attributed to either methodological differences or to a divergent behavior of the triazole-linkage in comparison to the isopeptide bond. At this point we note that changes in chemical shifts do not inevitably result in an apparent change of the SASA as they can also be caused exclusively by structural changes. This is assumed to be the case for the perturbations observed within the β_3_- and β_5_-strands comprising the proximal unit of artificially Lys27-linked Ub_2_ (Fig. 2B). Bearing this in mind, a distinct area on the molecular surface can be defined that equally shows up in CSP and ΔSASA analysis (Fig. S10B). Conclusively, this area on the surface of the proximal moiety is covered by the distal one in the solution averaged ensemble characterizing Lys27-linked Ub_2_. It comprises the central α-helix and the following loop region including the first 3_10_-helix. The orientation of the distal unit within Ub_2_ that one would deduce from these data is also capable to explain the strong CSP of Asp52 and the high ΔSASA value of Gly53 (Fig. 2B), respectively, because both residues are located in vicinity to the occupied surface area. We note that a CSP value is not available for Gly53, because its NMR peak is exchange broadened in the ^1^H-^15^N HSQC spectrum of UbK27C which serves as the reference spectrum for the calculation of CSP values.

Finally, CSP and ΔSASA value analysis has been performed for the canonical Lys63-linkage type. Although the distal moiety of such artificially Lys63-linked Ub_2_ does not induce major CSPs in the corresponding proximal moiety, it induces significant ΔSASA values (Fig. 2C). These computed values cluster especially in the unstructured region where the conjugation site is harbored as well as in the second 3_10_-helix and the β_2_/α_1_-loop which are in spatial proximity (Fig. 2C, S10C). Since both Ub units are rather unconstrained in case of the natural Lys63-linkage type, the corresponding dimers are capable to adopt multiple temporary conformations with transient domain-domain contacts^47^. Because the domain-domain orientation of Ub_2_ is apparently averaged in solution when using an analysis of chemical shifts only, a distinct set of overall conformations of Lys63-linked Ub_2_ is not detected by this method. In contrast, a decrease of SASA values is found in the analysis of collapsed conformations extracted from MD simulations. The large conformational variability of Lys63-linked Ub_2_ as proposed by the data obtained by NMR spectroscopy is underpinned by the fact that non-zero ΔSASA values are found widespread over the primary sequence (Fig. 2C).

### Domain-domain dynamics goes along with a broad conformational ensemble of Ub dimers

The conformational space which is occupied by polyubiquitin chains is based on the high flexibility of the linker connecting the two subunits. How does the position used for conjugating the two subunits in Ub_2_ controls this conformational ensemble? To address this question we performed NMR spectroscopic amide proton exchange experiments illuminating the proximal units of artificially Lys11-, and Lys27-linked Ub_2_s. This dynamic NMR experiment is capable to characterize potential domain-domain dynamics of those non-canonically conjugated chain types taking place on a millisecond time scale. By using a modified MEXICO approach, the exchange of exposed amide protons with solvent protons becomes apparent^48,49^. Since the distal moiety of Ub_2_ potentially hampers this exchange in the proximal one, insights into the domain-domain orientation between the subunits can be obtained complementing structural data gathered for both chain types so far. It has been shown recently that the modified MEXICO approach allows to reliably illuminate domain-domain conformation of artificially Lys48-linked Ub_2_^50^.

**Figure 3:**
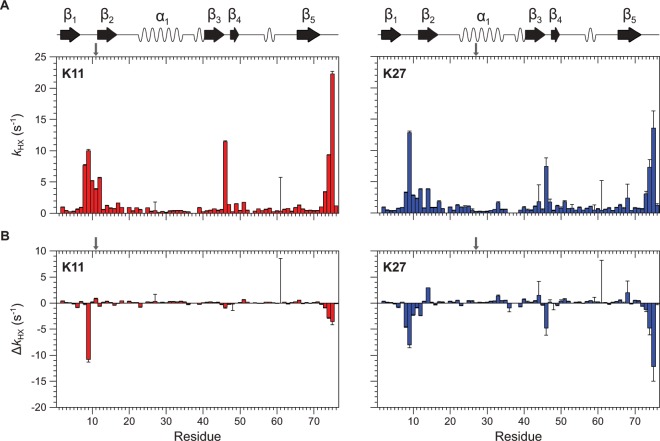
Exchange rate constants, *k*_HX_, obtained by using the modified MEXICO NMR experiment monitoring potential exchange between amide protons and solvent protons taking place on a millisecond time scale. (**A**) Data are shown for residues comprising the proximal unit of artificially PA-linked Ub_2_s originating from Lys11- (colored in red) or Lys27-linkage type (colored in blue). (**B**) Differences of amide proton exchange rate constants, Δ*k*_HX_, comparing data presented in A to the exchange between amide protons and solvent protons taking place in monomeric wild type Ub. Secondary structural elements according to PDB ID 1D3Z are indicated on top.

Due to the compact β-grasp fold of monomeric wild type Ub most of the residues show exchange rate constants less than 2 s^−1^ (at a pH value of 6.8) and cannot be reliably probed by this experiment^51^. This simplifies data analysis by focusing on residues located in less protected regions (Leu8-Thr12, Ala46, Leu73-Gly75) that are far apart from each other in sequence and represent suitable reporters^52–54^. Reliable amide proton exchange rate constants could be monitored for those residues mentioned above in both artificially Lys11-, and Lys27-linked Ub_2_s, respectively (Fig. S11, Table S1). The exchange rate constants characterizing domain-domain dynamics in Ub_2_s were shown to be slowed down compared to monomeric wild type Ub to a differing extent (Fig. 3A,B). Generally, the decrease of exchange rate constants is more pronounced for the Lys27- than for the Lys11-linkage type – correlating well with amplitudes of CSP values in corresponding structural data (Figs. 1C and 3A). Notably, the amide proton exchange data obtained for artificially Lys27-linked Ub_2_ reflect polar interactions between Arg72, Arg74, and Gly75 on the proximal moiety with Glu24, Glu51, and Asp52 on the distal moiety as it has been recently revealed in the three-dimensional structure of the corresponding natively isopeptide-linked Ub_2_ obtained by using x-ray crystallography^30^. Those domain-domain contacts lead to a pronounced decrease of exchange rate constants for residues close to the C-terminus (Leu73-Gly75) in the proximal unit of artificially Lys27-linked Ub_2_ compared to monomeric wild type Ub (Fig. 3B). A moderate decrease of the exchange rate constant of Ala46 which is spatially close to Glu51 and Asp52 can be monitored, too (Fig. 3A,B). Furthermore, both residues are located in a loop segment possessing residues showing strong CSP value (Asp52) and a significant loss of ΔSASA (Gly53) in consequence of their vicinity to the distal Ub_2_ unit (Fig. 2B). Neither changes of exchange rate constants at the C-terminal tail nor of Ala46 can be observed for the proximal unit of artificially Lys11-linked Ub_2_. Consequently, the dynamic data obtained for artificially Lys27-linked Ub_2_ here by using the MEXICO approach underline the features found in the three-dimensional crystal structure of the corresponding isopeptide-linked Ub_2_ determined by using x-ray crystallography^30^.

Contrary, the exchange rate constant seen for Thr9 present in the β_1_/β_2_-loop is reduced to a higher extent in the proximal unit of artificially Lys11-linked Ub_2_ compared to the Lys27-linked Ub_2_. Since that β_1_/β_2_-loop harbors the conjugation site used for Lys11-linkage, the distal moiety is restrained at the front of this loop in the proximal moiety of artificially Lys11-linked Ub_2_ preventing hydrogen exchange. This agrees well with the relative orientation of the two Ub_2_ subunits as it has been illuminated by means of CSPs and ΔSASA values (Fig. 2A).

In addition to the MEXICO approach outlined above, NMR diffusion methodology has been applied to shed further light on the domain-domain conformation of both artificially non-canonical Lys11-, and Lys27-linked Ub_2_s. Note that diffusion NMR spectroscopy has been used before as a potent tool to monitor the overall conformation of multi domain proteins^55,56^. The analysis of the translational diffusion profiles for artificially Lys11-, and Lys27-linked Ub_2_s obtained here leads to comparable diffusion coefficients of *D* = (8.95 ± 0.08) * 10^−11^ m^2^s^−1^ and *D* = (8.89 ± 0.04) * 10^−11^ m^2^s^−1^, respectively (Fig. S12A,B). For comparison, the diffusion coefficients of artificially conjugated Lys48-, and Lys63-linked Ub_2_s have been monitored as well and could be determined to *D* = (9.5 ± 0.1) * 10^−11^ m^2^s^−1^ and *D* = (9.2 ± 0.2) * 10^−11^ m^2^s^−1^ (Fig. S12C,D) indicating an apparent faster diffusion of this types of Ub_2_s compared to Lys11-, and Lys27-linked Ub_2_s. Note that structural investigations of both naturally and artificially Lys48-linked Ub_2_s have illuminated that dimers of this type of linkage adopt preferably a closed overall conformation^7,50^. The diffusion properties of Lys11-, Lys27-, and Lys48-linked Ub_2_s can thus be interpreted in such a way that the distal and proximal moieties of Lys48-linked Ub_2_ come more into close proximity in a time-averaged ensemble as is the case for Lys11-, and Lys27-linked Ub_2_s which show rather comparable hydrodynamic dimensions to each other. In other words, the diffusion data indicate that both Lys11-, and Lys27-linked Ub_2_s are less compact than Lys48-linked Ub_2_. Moreover, the diffusion data shows that artificially conjugated Lys63-linked Ub_2_ diffuses slower compared to the Lys48-linked counterpart suggesting a less compact conformation consistent with observations done for isopeptide-linked Ub_2_s using these sites for the linkage^11^. However, our diffusion data also indicate that Lys11-, and Lys27-linked Ub_2_s are apparently slightly larger in hydrodynamic dimension than Lys63-linked Ub_2_.

Coarse grained simulations have been complementary used to probe the size of Lys11-, Lys27-, Lys48-, and Lys63-linked Ub_2_s. From the long time scale CG simulations (120 µs per linkage) we have computed the mean radius of gyration, *r*_G_. Note that here the hydration shell around Ub_2_ is not included in the estimation of *r*_G_ and can therefore not be compared to the NMR data in a quantitative manner. However, it has been previously shown that relative differences between experimentally measured *D* values can be reproduced by comparing with values obtained for *r*_G_^56^. For the present study, one should also point out that although frequent transitions between different open and compact conformations are found in the coarse grained simulations for all linkage types, open conformations might be systematically underrepresented in the model and weights between different states might be still not converged to a full extent. Thus the computed *r*_G_ should be taken with a grain of salt. Nevertheless, the trend observed here for computed values of *r*_G_ when comparing the four linkage types is not in conflict with the experimental results made for *D*. Lys48-linked Ub_2_ shows the most compact conformation with *r*_G_^Lys48^ = (16.8 ± 1.4) Å. The dimensions of Lys11-, Lys27-linked Ub_2_s, *r*_G_^Lys11^ = (17.2 ± 1.2) Å, *r*_G_^Lys27^ = (17.9 ± 1.2) Å, are comparable or rather larger than the dimension determined for Lys63-linked Ub_2_ possessing *r*_G_^Lys63^ = (17.5 ± 1.5) Å, respectively.

### Illuminating linkage-specific backbone dynamics in Ub dimers

**Figure 4:**
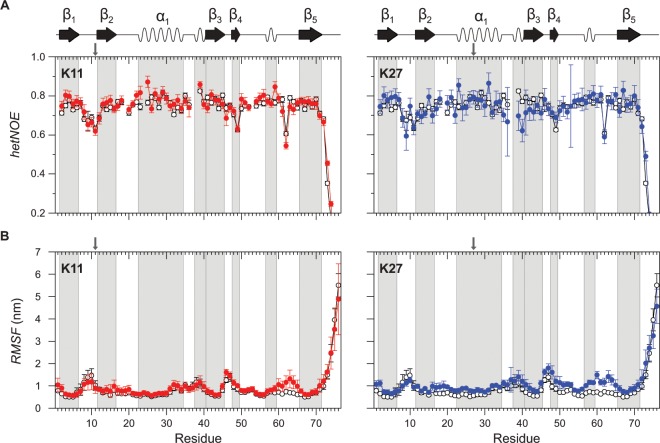
(**A**) {^1^H}-^15^N heteronuclear NOEs (*hetNOE*) determined by NMR spectroscopy in comparison to (**B**) simulated root mean square fluctuations (*RMSF*) obtained by MD simulations. *HetNOE* values in (**A**) are measured for residues comprising the proximal moiety of artificially PA-linked Ub_2_s originating from Lys11 (colored in red, left) and Lys27 (colored in blue, right), whereas *RMSF* values in (**B**) are calculated for residues comprising the proximal moiety of natively isopeptide-linked Ub_2_s also originating from Lys11 (colored in red, left) and Lys27 (colored in blue, right). Corresponding data from monomeric wild type Ub are colored in white. Secondary structural elements according to PDB ID 1D3Z are indicated on top and highlighted by using a background colored in gray.

In order to extend the dynamic view on non-canonically Lys11-, and Lys27-linked Ub_2_s to a faster time scale data from NMR spin relaxation and MD simulations have been acquired. We determined ^15^N-based *hetNOE* values for each residue comprising the proximal unit of artificially PA-linked Ub_2_s as well as *RMSF* values computed from the simulations of each residue comprising the proximal unit of corresponding natively isopeptide-linked Ub_2_s (Fig. 4A,B). Both parameters refer to motions of the ^1^H-^15^N bond vector on the picosecond to nanosecond time scale providing general information about backbone flexibility^57^. Generally, *hetNOE* values are high and *RMSF* values are low consistently over the sequence for both the Lys11-, and the Lys27-linkage type underlying the stability of the β-grasp Ub fold (Fig. 4A,B). This agrees well with the poor ability for amide to solvent proton exchange as it has been observed for most residues using the MEXICO experiment (Fig. 3A). Only regions already known for increased backbone dynamics - that are the β_1_/β_2_-loop and the C-terminal tail^36^ - exhibit low *hetNOE* as well as high *RMSF* values confirming the conservation of those dynamic features for both linkage types compared to monomeric wild type Ub (Fig. 4A,B). *RMSF* values are additionally increased for the proximal unit of both Lys11-, and Lys27-linked Ub_2_s compared to monomeric wild type Ub for residues in the unstructured region between the second 3_10_-helix and the β_5_-strand (Fig. 4B). We note that the increased flexibility in this region is linkage-independent and not confirmed by experimental NMR data (Fig. 4A). However, one significant difference comparing the proximal units of artificially Lys11-, and Lys27-linked Ub_2_s is apparent by analyzing the individual *hetNOE* values. A cluster of residues in the proximal unit of the artificially Lys27- but not Lys11-linked Ub_2_ shows decreased *hetNOE* values compared to monomeric wild type Ub. This cluster of residues displays the region between the central α-helix and the β_3_-strand including the first 3_10_-helix (Fig. 4A) which serves as a hotspot for the orientation of the distal moiety within the Lys27-linked Ub_2_ based on CSP and ΔSASA values (Figs. 2B, S10B).

In addition to *hetNOE* and *RMSF* values discussed above, the ^15^N relaxation rate constants *R*_1_ and *R*_2_ for residues comprising the proximal units of both the artificially Lys11-, and Lys27-linked Ub_2_ have been determined as well to study fast internal motions even further (Fig. S13A,B). Due to the difference in the rotational correlation time taking monomeric Ub and Ub_2_ species into account, the relaxation rate constants of Ub_2_s are not directly comparable with those obtained for monomeric wild type Ub. However, similar *R*_1_ values of both the artificially Lys11-, (*R*_1_^mean^ = 1.3 ± 0.1 s^−1^) and the Lys27-linked Ub_2_ (*R*_1_^mean^ = 1.3 ± 0.1 s^−1^) (Fig. S13A) suggest that these dimers experience comparable contributions from anisotropic molecular rotation. This is strongly underpinned by the results obtained from independent NMR diffusion experiments assuming a compact conformation in solution for both type of linkages possessing comparable dimensions as reflected in coinciding diffusion coefficients (Fig. S12A,B). Consequently, the *R*_2_/*R*_1_ value is a reliable estimate for the rotational correlation time of molecules and thus can be used to identify potential linkage-specific differences in the dynamic behavior of the Ub_2_s on the fast picosecond to nanosecond time scale^58,59^. For this purpose we determined individual *R*_2_/*R*_1_ values for each residue present in the proximal unit of artificially Lys11-, and Lys27-linked Ub_2_s and calculated the 10% trimmed mean as an average value for both type of linkages^60^. Contributions to the relaxation of residues undergoing large amplitude of motions or exhibiting chemical exchange can be excluded in this way. Variations in individual *R*_2_/*R*_1_ values differing more than one standard deviation from the 10% trimmed mean can thus be interpreted as additional internal motions that are either faster or slower than the overall rotational correlation time of the whole molecule^60^.

In case of Lys27-linked Ub_2_ *R*_2_/*R*_1_ values are elevated especially for residues at the C-terminal end of the central α-helix and the β_3_-strand (Fig. S13C) indicating reduced dynamics within those elements on the picosecond to nanosecond time scale. Besides, Gly53 and Val70 that exhibit extremely large *R*_2_/*R*_1_ values show high *R*_2_ values simultaneously (Fig. S13B,C). Conclusively, these two residues presumably exhibit contributions from chemical exchange suggesting changes in dynamics also on the slower microsecond to millisecond time scale. This is amplified by the fact that the NMR cross signal of Gly53 (and also of Glu24 which is spatially close) is usually exchange-broadened in the two-dimensional ^1^H-^15^N HSQC spectrum of monomeric wild type Ub but reappears during the dimer formation procedure of artificially Lys27-linked Ub_2_. Dynamics on this time scale can thus be attributed to local structural rearrangements as described in the first section. This is congruent with reportings from the corresponding natively isopeptide-linked Ub_2_^27^. In contrast to the Lys27-linkage type, *R*_2_/*R*_1_ values of residues comprising the proximal unit of artificially Lys11-linked Ub_2_ are rather widespread around the average (Fig. S13C). Clusters of residues with reduced values indicating fast fluctuations on the picosecond to nanosecond timescale are observed in the β_2_/α_1_-loop and around the second 3_10_-helix in the unstructured region between the β_4_- and β_5_-strands, whereas a number of residues in the α-helix has elevated values and is thus slowed down (Fig. S13C).

**Figure 5:**
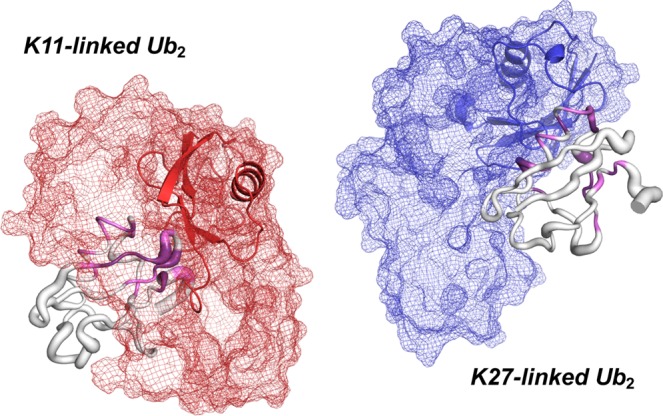
Proposed conformations of naturally isopeptide-linked Ub_2_s originating from non-canonical Lys11- (colored in red, left), and Lys27-linkage (colored in blue, right). These two structures have been obtained from an ensemble of simulated Ub_2_ conformations lowest in free energy by using a combination of the experimental and simulated data presented in this study. The proximal Ub_2_ moieties are colored in light gray whereas the distal moieties are colored in red (in case of Lys11-linkage) and in blue (in case of Lys27-linkage), respectively. Individual *R*_2_/*R*_1_ values obtained for the proximal Ub_2_ moieties (Figs. S13C, S14B) indicating backbone dynamics on the fast NMR time scale are shown in putty mode. Values lower than the 10% trimmed mean are shown in thick mode, values larger than the 10% trimmed mean are shown in thin mode in respective proximal moieties (continuous scale). Chemical shift perturbations shown in Fig. 1C are highlighted by using violet color for Δω values larger the mean and dark purple color for Δω values larger the mean plus one standard deviation. The conformational space covered by the distal Ub_2_ moiety obtained from MD simulations is illustrated in mesh mode and is based on four representative structures of the dominant minima in free energy found in the coarse grained simulations. The configuration of Ub_2_ that fits best to the experimentally obtained NMR data is highlighted by presenting the distal moiety in cartoon mode. The structures have been created by using the PyMOL Molecular Graphics System, Version 2.4.0a0, Schrödinger, LCC (www.pymol.org).

It becomes obvious that for both non-canonical linkage types, Lys11 and Lys27, residues in the central α-helix of the proximal moiety become rigid on the picosecond-to-nanosecond time scale upon adding the distal unit (Fig. S14A,B). This part in the proximal unit of Ub_2_ is partially constrained by the presence of the distal moiety seen in the structural model developed for both linkage types based on the experimentally and simulated data obtained in this study (Fig. 5). On the basis of *R*_2_/*R*_1_ values we thus suggest that fast time scale dynamics are generally slowed down for residues residing at the domain-domain interface when the proximal moieties get in contact with the distal ones. Furthermore, in case of artificially Lys11-linked Ub_2_, residues of the proximal moiety showing increased fast time scale dynamics (the β_2_/α_1_-loop and the second 3_10_-helix) are located on the side which is supposed to be averted from the distal moiety (Fig. 5). In contrast, in the case of artificially Lys27-linked Ub_2_ residues comprising the first 3_10_-helix of the proximal moiety representing the contact site with the distal one possess increased fast time scale dynamics (verified by *hetNOE* relaxation data), whereas the succeeding β_3_-strand exhibits reduced dynamics although it is presumably not in contact with the distal moiety (Fig. 5). However, since both elements in the proximal Ub_2_ moiety adjoin the conjugation site of the Lys27-linkage their dynamics is rather affected by structural disturbance than an interaction with the distal moiety. In summary, fast time scale dynamics in the proximal moiety of both artificially Lys11-, and Lys27-linked Ub_2_s are modulated in a way that motion is either slowed down at the interdomain contact site or accelerated at the exposed site.

### Probing the ability of artificially conjugated Ub dimers to interact with ligands

NMR titration experiments have been performed to probe the ability of artificially conjugated Ub_2_ to interact with potential binding partners. We have focused on monitoring the interaction of the ubiquitin associated (UBA) domain 2 from Rad23 to artificially Lys11-, Lys27-, and Lys63-linked Ub_2_s as it has been shown that this UBA domain binds to naturally linked Ub_2_s by possessing these different sites used for conjugation^11,27,29^.

**Figure 6:**
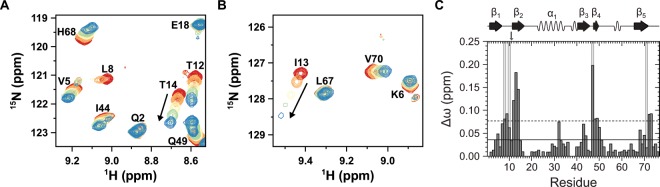
Functional characterization of artificially Lys11-linked Ub_2_. (**A**,**B**) Close-up views of selected cross signals in two-dimensional ^1^H-^15^N HSQC spectra following the interaction of UBA2 from Rad23 with Lys11-linked Ub_2_ on the basis of isotopically labeled residues comprising the proximal moiety in this dimer. The increasing stoichiometry, *n*, of UBA2 regarding Lys11-linked Ub_2_ has been visualized by using a color coding ranging from red (*n* = 0), to orange (*n* = 0.3), to yellow (*n* = 0.9), to green (*n* = 1.7), and finally to blue (*n* = 3.1). The pronounced change in the chemical shift of I13 and T14 has been additionally highlighted by an arrow. (**C**) Overall change in chemical shifts of residues comprising the proximal moiety in Lys11-linked Ub_2_ comparing absence with presence of a three times excess of UBA2 regarding Ub_2_. The horizontal lines indicate Δω values larger than the mean (continuous mode) and larger than the mean plus one standard deviation (dotted mode). Residues undergoing a signal attenuation larger than 75% are highlighted by using a background colored in gray. Secondary structural elements according to PDB ID 1D3Z are indicated on top and the site used for conjugation has been highlighted by using a vertical arrow. Data for titrations of UBA2 to artificially Lys27-, and Lys63-linked Ub_2_s are shown in Fig. S16.

Thus non-isotopically labeled UBA2 from Rad23 has been stepwise added to Lys11-linked, Lys27-linked, and Lys63-linked Ub_2_s in which residues comprising the proximal domain have been ^15^N-enriched enabling the acquisition of a series of two-dimensional ^1^H-^15^N HSQC spectra. As a result, the addition of a three times stoichiometric excess of UBA2 to artificially Lys11-linked Ub_2_ leads to profound changes in chemical shifts, *Δω*, of proximal residues mainly located in the hydrophobic patch of Ub close to Leu8, Ile44, and Val70 possessing a maximum in *Δω* of about 0.2 ppm (Fig. 6). The regression of Eq. (5) to individual titration profiles illuminates an affinity of UBA2 to artificially Lys11-linked Ub_2_ of about *K*_D_ = (90 ± 40) *μ*M (Fig. S15A) nicely matching *K*_D_ = (155 ± 22) *μ*M and *K*_D_ = (197 ± 30) *μ*M seen for hHR23A-UBA2 interaction to residues comprising the distal or proximal moiety in naturally Lys11-linked Ub_2_, repectively^29^. We have extended the functional characterization of artificially conjugated non-canonical Ub_2_s by performing an NMR spectroscopic based titration experiment of adding UBA2 to Lys27-linked Ub_2_. Here, presenting a 2.4 times stoichiometric excess of unlabeled UBA2 to Ub_2_ results in changes of chemical shifts and a significant decrease of the signal intensity of similar residues which have been observed for adding of UBA2 from hHR23A to the naturally counterpart (Fig. S16A)^27^. Quantitatively, artificially Lys27-linked Ub_2_ recognizes the UBA2 domain with an affinity of about *K*_D_ = (270 ± 130) *μ*M (Fig. S15B) which is slightly increased compared to *K*_D_ = (42 ± 8) *μ*M and *K*_D_ = (63 ± 17) *μ*M reported for the recognition of hHR23A-UBA2 by the proximal or distal ubiquitin moiety present in naturally Lys27-linked Ub_2_^27^. Finally, Lys63-linked Ub_2_ has been additionally used to shed light on the general ability of ligand recognition done by Ub_2_s which have been assembled by using an artificial triazole linkage. Adding a 4.6 times stoichiometric excess of UBA2 regarding artificially Lys63-linked Ub_2_ induces changes in chemical shifts which are highest for residues Ile13, Gly47, Leu71, and Leu73 possessing *Δω* values of about (0.08 … 0.1) ppm (Fig. S16B). Note that in naturally Lys63-linked Ub_2_ the same residues get affected when hHRA23A-UBA2 domain is added by analyzing changes in chemical shifts^11^. Quantitatively, the affinity between UBA2 and artificially Lys63-linked Ub_2_ can be determined to *K*_D_ = (80 ± 10) *μ*M (Fig. S15C) which is comparable with the affinity seen for UBA2 interaction to distal or proximal ubiquitin present in naturally Lys63-linked Ub_2_ which has been reported with *K*_D_ = (280 ± 100) *μ*M and *K*_D_ = (180 ± 80) *μ*M, respectively^11^.

To conclude, both artificially conjugated non-canonical as well as canonical Ub_2_s are fully capable to recognize binding partners. The binding sites present in artificially conjugated Ub_2_s and the affinity seen for the UBA2-Ub_2_ interaction presented here resemble results which have been reported for the naturally linked counterparts. This result strongly underlines the potent reliability of the artificial conjugation used for the assembly of Ub_2_s beside the structural and dynamical performance probed complementary.

### Verifying domain-domain conformation of artificially conjugated Ub dimers

The efforts done for the conformational characterization of artificially conjugated non-canonical Ub_2_s presented in this manuscript converge into representative conformations of Lys11-, and Lys27-linked Ub_2_s which have been derived from both NMR spectroscopic data and MD simulations, respectively (Fig. 5). The proximal moiety is displayed such that the key NMR data (CSP values which hint at closeness of the distal moiety and *R*_2_/*R*_1_ values pointing out dynamical features) are displayed by color and thickness of the ribbons. The distal moiety is displayed as a superposition of four conformations from the MD ensemble (free-energy minima from the CG and subsequently back-mapped MD simulation). The domain-domain orientation between the proximal and the distal Ub_2_ unit which best represents the NMR data has been highlighted in Fig. 5 using a cartoon mode presentation. In this way it can be seen that in order to fully account for the NMR data, e.g. the observed CSPs, the full extent of the conformational ensemble, i.e. more than only one of the representatives from the simulations, is required. Since for both linkages the proximal moieties have been arranged in the same orientation, Fig. 5 also nicely illustrates that the coverage of the surface of the proximal moiety is completely complementary in the Lys11-, and Lys27-linked Ub_2_s.

The three-dimensional structural ensemble of artificially Lys11-linked Ub_2_ obtained here by combining NMR spectroscopy with MD simulations (Fig. 5, left) enables a comparison with available structures of Ub_2_ possessing the same site of linkage conjugated using an isopeptide bond (Fig. S17). The structures used for this comparison are based on experimental data obtained by NMR spectroscopy (Fig. S17A,B)^29^ and by using X-ray crystallography (Fig. S17C,D)^19,28^. The favored conformation of artificially Lys11-linked Ub_2_ – for that the distal moiety has been colored in red in Fig. S17 - fits best to 2MBO and 2MBQ both derived by NMR spectroscopy (Fig. S17A,B). This result indicates that, firstly, the triazole-linkage used here operates even for non-canonical conjugation as a fully reliable surrogate for isopeptide-linked Ub_2_s besides for the already shown canonical type of linkage^50^. Secondly, it confirms that the profound combination of high-resolution NMR spectroscopy and MD simulations presented here indeed allows to get structural and dynamical insights into domain-domain conformations of Ub_2_s in a solution averaged ensemble avoiding individual isotopic enrichment of both moieties comprising Ub_2_s. Note that the crystal structure 2XEW representing isopeptide Lys11-linked Ub_2_ is covered by the conformational landscape which has been computed in our study for this site of linkage as well as shown in Fig. S17C. Consequently, we conclude that the crystallization competent conformation included in 2XEW inherently belong to the conformational landscape of Lys11-linked Ub_2_. Finally, the structural ensemble of Lys11-linked Ub_2_s computed in our study and shown in Fig. 5, left and Fig. S17 is not in significant conflict with the conformation shown in 3NOB (Fig. S17D) determined by using X-ray crystallography. We conclude that the beneficial combination of NMR spectroscopy with MD simulations presented here enables the precise determination of the conformational space Lys11-linked Ub_2_s occupy in a solution averaged ensemble to fully account for the inherent flexibility this type of linkage possesses.

## Conclusions

In summary, we have been able to successfully generate non-canonically conjugated Ub_2_s based on a semisynthetic approach in milligram quantities and high purity. This strategy impressively shows the large potential of using non-native linkages for the synthesis of Ub_2_s which allows to subsequently conduct highly resolved NMR spectroscopic experiments distinctly probing one of the Ub_2_ moieties at atomic resolution. In this way we used a comprehensive experimental strategy to extract the structural, dynamical, and functional features of these Ub conjugates on a residue-by-residue basis. As an important result, non-natively linked Ub_2_s mirror the natively linked counterparts very reliably in terms of structural as well as dynamic and functional properties and the artificial linkage used here acts as a valid surrogate for isopeptide-linked Ub_2_s. NMR spectroscopic and MD simulations data have precisely illuminated how the proximal moiety of Ub_2_s gets affected when it is linked with the distal counterpart and to what extent the position used for domain-domain linkage accurately controls this property. This is of particular interest for the Lys27-linkage where the conjugation process disturbs the inherent role of the lysine side chain in the native Ub fold. Computationally, we have been able to extract low free-energy conformations of Ub_2_s from long-time scale, comprehensive CG simulations. These data have been back-mapped to perform simulations on an atomically resolved level. Thus representative structures of a well equilibrated ensemble of domain-domain conformations could be obtained. As the key result we have developed a structural model which is based on experimental and computational efforts depicting the conformational ensemble for the two non-canonically conjugated Ub_2_s based on Lys11-, and Lys27-linkage present in solution (Fig. 5). For both linkages, four representative configurational states of Ub_2_s have been taken into account and it was found that this structure bundle agrees very well with the structural and dynamical results obtained from NMR spectroscopy. Summarizing, in the case of the Lys11-linked Ub_2_ the distal moiety mainly covers the β-sheet part of the proximal chain whereas in the case of the Lys27-linked Ub_2_ the distal unit covers the α-helical part of the proximal moiety. This difference between Lys11-, and Lys27-linked Ub_2_s seen in the conformational ensembles indicates that Ub_2_s adopt characteristic ensembles of multiple stable conformations in thermodynamic equilibrium in solution which may play a crucial role for linkage specific interactions with potential binding partners. Indeed, the role of linkage specific interactions has been shown here by the different recognition of the UBA2 domain done by Lys11-, Lys27-, and Lys63-linked Ub_2_s. This supports the notion that the inherent structural and dynamical features of different Ub_2_s is the basis of their linkage specificity which finally cause dissimilar cellular functionalities. Finally, the approach presented here by combining NMR spectroscopy with MD simulations applied on Ub_2_s may pave the way for the in-depth characterization of other multidomain molecules present in biology.

## Methods

### Expression and purification of Ub monomers

All plasmids used for separate expression of distal and proximal Ub_2_ moieties were kindly provided by X. Zhao (Rockefeller University, USA) whereas ^15^N-isotopically labeled monomeric wild type Ub was purchased from Giotto Biotech (Italy). The distal unit UbG75Aha devoids C-terminal Gly76 and bears the unnatural amino acid l-azidohomoalanine (Aha) instead of Gly75. This is accomplished by selective pressure incorporation since the glycine codon at that position in the DNA sequence is replaced by a methionine codon. In addition, the N-terminus is equipped with a GST-tag and thrombin cleavage site and lacks the initial methionine to avoid an alternative incorporation site^32,61^. The proximal units UbK11C, UbK27C and UbK63C are single mutants of Ub with a cysteine residue in place of the lysine at the desired linkage position ensuring site-directed dimer conjugation^32^.

A methionine auxothropic *E. coli* B834 (DE3) strain (Novagen) with corresponding pGEX2TK vector (GE Healthcare) was used for overexpression of unlabeled UbG75Aha. A preculture was grown at 37 °C in New Minimal Medium (NMM) supplemented with 100 mg/l carbenicillin (Carl Roth) and 50 mg/l of all natural proteinogenic amino acids (Sigma-Aldrich), except of methionine in a limiting concentration of 0.05 mM. At an OD_600_ of ≈1.3 bacteria were spun down and resuspended in fresh NMM containing no methionine anymore, but Aha (Iris Biotech) in a concentration of 0.5 mM. After 30 min incubation at 37 °C, protein expression was induced by addition of 1 mM IPTG (Carl Roth) and performed overnight at 25 °C. The cell pellet was harvested by centrifugation, resuspended in PBS buffer (pH 7.3) with 1% (v/v) Triton X-100 (Carl Roth) and lysed by sonication. Cell debris were removed by centrifugation and GST-tagged Ub from the supernatant was permitted to bind to glutathione sepharose beads (GE Healthcare) for 6 h at 4 °C. Then the beads were poured into a column, washed with PBS buffer and the tag was cleaved by human thrombin (Sigma-Aldrich) overnight at room temperature. The protein solution was finally applied to a HiLoad 16/600 Superdex 75 pg column (GE Healthcare) for size exclusion chromatography (SEC) and pure fractions were concentrated and frozen at −20 °C. Unlabeled UbG75Aha could be generated with about 2.9 mg per litre expression culture.

Uniformly ^15^N-labeled UbK63C and ^13^C/^15^N-labeled UbK11C and UbK27C were overexpressed in *E. coli* BL21(DE3) cells (Invitrogen) from pET3a vectors (Novagen). Bacteria were grown in M9 minimal medium with 100 mg/l carbenicillin and either ^15^NH_4_Cl (Cortecnet) or additionally ^13^C-D-Glucose (Cambridge Isotope Laboratories) as the sole sources of nitrogen and carbon, respectively^62,63^. At an OD_600_ of 0.6–0.7 1 mM IPTG was added and protein expression was performed overnight at 25 °C. After harvesting by centrifugation, cells were resuspended in 20 mM NaOAc buffer (pH 4.5), lysed by sonication and spun down again. Thermolabile components were removed by heat precipitation and subsequent centrifugation and the supernatant was purified further by cation exchange chromatography using a HiTrap SP HP column (GE Healthcare) with a 1 M NaCl gradient. Ub containing fractions were pooled, concentrated and reduced with 20 mM TCEP (Sigma-Aldrich) prior to SEC (see above) with an elution buffer consisting of 25 mM Tris, 300 mM NaCl and 2 mM TCEP (pH 7.5). Pure fractions were concentrated and transferred into 20 mM Tris buffer (pH 7.5) and were directly used for dimer formation and NMR experiments. We have been able to generate 2.2 mg (doubly ^13^C/^15^N labeled monomeric UbK11C), 3.1 mg (doubly ^13^C/^15^N labeled monomeric UbK27C), and 13.3 mg (singly ^15^N labeled monomeric UbK63C) per litre expression culture, respectively.

### Bioorthogonal Ub dimer formation

Ub_2_ formation is implemented by a site-specific reaction of propargyl acrylate (PA) with the thiol group of the cysteine present in the proximal Ub_2_ unit followed by a bioorthogonal click reaction using the azide functionality of the Aha side chain present in the distal unit to form a triazole-linkage (Fig. S1)^32^. First, cysteine mutated Ub was diluted to a protein concentration of 100 µM with 20 mM Tris buffer (pH 7.5) and were then reduced with 50 mM TCEP to ensure accessibility and reactivity of free thiol groups. The linker reaction was initiated at a protein concentration of 20 µM by addition of a 200-fold molar excess of PA (Sigma-Aldrich) solved in the same buffer but supplemented with 10% (v/v) MeCN (Riedel-de Haen) and executed under rigorous shaking at 25 °C. Small samples were taken at regular time steps and reacted with a 25-fold molar excess of fluorescein-5-maleimide (Tokyo Chemical Industry) to monitor the reaction process on SDS-PAGE under UV light. Complete consumption of free thiol groups in Ub was detected by vanishing fluorescence and excess PA was removed by gradient dialysis at 4 °C. This part has been performed in three steps lasting 12 h each against 20 mM Tris buffer (pH 7.0) with decreasing amounts of 50%, 20% and 0% (v/v) MeOH (Sigma-Aldrich), respectively. The protein solution was concentrated and, if necessary, subjected to size exclusion chromatography again (see above).

Ub_2_s were formed via the Cu(I)-catalyzed azide-alkyne cycloaddition reaction for 1 h under argon atmosphere and on ice. The reaction solution contained 100 µM of both Ub_2_ moieties in 20 mM Tris buffer (pH 7.5) supplemented with 0.5 mM SDS (Carl Roth), 6 mM THPTA (Sigma-Aldrich) and 3 mM Cu(MeCN)_4_BF_4_ (Sigma-Aldrich). Unreacted monomer species were removed by SEC (see above) and fractions of pure Ub_2_ were combined, concentrated and directly used for NMR measurements. Chromatograms for Lys11- and Lys27-linked Ub_2_s obtained by running SEC are shown in Fig. S18 which are accompanied by SDS-PAGE analyses. All protein concentrations in this study were determined by BCA assay (Thermo Scientific).

Lys48-linked Ub_2_ used for the determination of the diffusion coefficient has been prepared as described before^50^.

### Expression and purification of Rad23-UBA2

Rad23-UBA2 (sequence 277–323; Gene ID 174785) was recombinantly expressed in *E. coli* BL21 Rosetta (DE3) cells (Novagen) as a 6xHis-SUMO fusion protein. Cells were grown to an OD_600_ of 0.6 at 30 °C and protein expression was induced by addition of 1 mM IPTG for 4 h at 30 °C. Cells were harvested by centrifugation, snap-frozen in liquid N_2_ and stored at −80 °C. Cell pellets were thawed at room temperature and resuspended in ice cold lysis buffer (50 mM Na_3_PO_4_ (pH 8), 300 mM NaCl, 6 mM MgCl_2_, 10% (v/v) glycerol, 2 mM β-mercaptoethanol) containing 10 µg/ml DNAse I (Sigma-Aldrich), 2 mM phenylmethysulfonyl fluoride (Carl Roth), 10 µg/ml aprotinin (Genaxxon), 8 µg/ml pepstatin A (Genaxxon) and 5 µg/ml leupeptin (Genaxxon). After resuspension cells were lysed by French press and the lysate cleared by centrifugation. The supernatant was incubated with Ni^2+^-iminodiacetic acid resin (Machery-Nagel) and loaded on a gravity flow column. The column was washed with lysis buffer containing 750 mM NaCl (without DNAse and protease inhibitors) and additionally with lysis buffer containing 25 mM NaCl. Protein was eluted with lysis buffer containing 250 mM imidazole (pH 8) (Merck). The elution fraction was dialyzed in ion exchange buffer (20 mM Na_3_PO_4_ (pH 7.5), 25 mM NaCl, 6 mM MgCl_2_, 10% (v/v) Glycerol, 2 mM β-mercaptoethanol) overnight at 4 °C and loaded on a Resource Q 6 ml anion exchange chromatography column (GE Healthcare) using a 650 mM NaCl gradient. Elution fractions containing the fusion protein were pooled, the 6xHis-SUMO tag was cleaved overnight at 4 °C using 24 µg Ulp1 protease (in-house purification) per mg fusion protein, followed by tag removal using Ni^2+^-iminodiacetic acid resin. Rad23-UBA2 fractions were collected and the purity was checked on a Coomassie-stained SDS-PAGE gel. Protein was concentrated in a dialysis tubing with 1 kDa molecular weight cut-off on a Spectra/Gel Absorbent (Spectrum Laboratories) at 4 °C, dialyzed into 20 mM Na_3_PO_4_ (pH 6.8) and stored at −80 °C.

### NMR Sample preparation and assignment of chemical shifts

Samples of cysteine mutated monomeric Ubs with and without PA linker were prepared in 30 mM MOPS buffer with 50 mM NaCl and 5% (v/v) D_2_O (pH 6.8), supplemented with 10 mM TCEP in presence of free thiol groups. Ub_2_ samples were buffered in 20 mM Na_3_PO_4_ and 5% (v/v) D_2_O (pH 6.8). All NMR experiments were performed on an Avance III 600 MHz spectrometer (Bruker) equipped with either a quadrupole (QXI) resonance room temperature probe or triple (TCI) resonance cryo probe at *T* = 298 K. Datasets were processed using NMRPipe^64^ and analyzed with NMRView^65^.

Backbone resonances of ^15^N singly labeled UbK63C with and without PA linker were assigned by three-dimensional (3D) ^15^N TOCSY-HSQC (80 ms mixing time) and 3D ^15^N NOESY-HSQC spectra (120 ms mixing time). Due to moderate peak shifts in case of the Lys63-linkage type, assignments based on the monomeric species could be transferred unambiguously to the corresponding peaks representing Ub_2_ species with assistance of a 3D ^15^N NOESY-HSQC spectrum (90 ms mixing time). ^1^H, ^15^N backbone resonances of ^13^C/^15^N doubly labeled UbK11C and UbK27C were verified by the triple resonance experiments HNCA, HNCO and HN(CO)CACB. HNCA experiments have also been acquired for the assignment of monomeric Ub possessing PA linker and subsequently of corresponding Ub_2_.

Differences in chemical shifts were calculated according to the following equation^66^:

1 $$\Delta \omega =\sqrt{\frac{{({\Delta }^{1}H)}^{2}+\frac{1}{25}{({\Delta }^{15}N)}^{2}}{2}},$$

where Δ^1^H is the change in proton and Δ^15^N is the change in nitrogen dimension, respectively, between corresponding peaks.

### Monitoring exchange of amide protons

A modified version of the MEXICO experiment (measurement of fast proton exchange rates in isotopically labeled compounds) based on ^1^H-^15^N HSQC spectra was used to obtain dynamic information in the millisecond time regime^48^. Rate constants of hydrogen exchange with the solvent were individually determined for each amide proton comprising the proximal moiety of Lys11- and Lys27-linked Ub_2_s as well as monomeric wild type Ub. Peak intensities were detected at different exchange periods ranging from 10 to 250 ms and were used for fitting using the following double-exponential function^49^:

2 $$S=(\frac{{k}_{{\rm{HX}}}}{{R}_{1}+{R}_{1{\rm{w}}}})({e}^{-{R}_{1{\rm{w}}}t}-{e}^{-({R}_{1}+{k}_{{\rm{HX}}})t}),$$

where *S* is the signal intensity relative to the reference ^1^H-^15^N HSQC spectrum, *k*_HX_ is the rate constant of proton exchange and *R*_1_ is the longitudinal relaxation rate constant of individual amide protons. The relaxation rate constant *R*_1w_ of water protons was separately determined to 0.31 s^−1^. Error values were estimated from the mean standard deviation of replicate measurements at two different exchange periods and were included in weighted curve fitting.

### ^15^N spin relaxation measurements

Backbone amide ^15^N longitudinal (*T*_1_) and transversal relaxation experiments (*T*_2_) were performed for the proximal unit of Lys11- and Lys27-linked Ub_2_s as well as for monomeric wild type Ub. Relaxation delay times were in a range of 10 to 3000 ms and 8 to 296 ms, respectively, to read out peak intensities for determination of individual *T*_1_ and *T*_2_ relaxation times. The peak intensities were fitted using the following single exponential equation:

3 $${I}_{t}={I}_{0}{e}^{-\frac{t}{{T}_{1,2}}},$$

where *I*_t_ is the peak intensity using a relaxation delay time *t* and *I*_0_ is the peak intensity omitting a relaxation period. Error values were calculated as described for amide proton exchange. The recycling delay between successive scans has been set to one second. The temperature of the sample kept – to the best of our knowledge – constant at *T* = 298 K as we have not observed any changes in the lock level of the spectrometer during the course of the experiment and as we have used an interpulse delay of about 900 *μ*s between successive 𝜋-pulses to follow the duty cycle defined by the probe.

The same samples were used for determining the {^1^H}-^15^N steady state NOE value (*hetNOE*) based on the ratio of the average peak intensity measured with or without proton saturation^67^. The mean standard deviation from two independent measurements was denoted as error value. The *hetNOE* experiment has been conducted in an interleaved fashion with alternating saturated and unsaturated transients. In this way the same conditions for both experiments were guaranteed despite the long measurement period. We have used a recycle delay of 3 s between successive scans. The saturation of protons has been made by using hard pulses of 120 degree for about 3 s (600 pulses have been applied separated by 5 ms each). Note that we have acquired one-dimensional proton NMR spectra permanently in between the determination of *T*_1_, *T*_2_ and *hetNOE* values making sure that these spectra remain constant over days in terms of signal intensity, linewidth and chemical shifts. This has been the case.

### NMR diffusion measurement

NMR diffusion spectra have been acquired at the proton dimension by using pulsed field bipolar gradient stimulated echo experiments at *T* = 298 K. For each diffusion profile, 21 different gradient strengths *G* were used for 6 ms along the z axis followed by a 100 ms recovery delay. The diffusion of Lys11- (*c* = 200 *μ*M), Lys27- (*c* = 185 *μ*M), Lys48- (*c* = 65 *μ*M), and Lys63-linked Ub_2_s (*c* = 35 *μ*M) was allowed to proceed for 100 ms. The calibration of *G* was performed by a standard protocol^68^. For error estimation, four different gradient strengths were repeated (relative gradient strengths of 1, 10, 40, and 70%). The measured ^1^H NMR spectra were integrated within the aliphatic signal region *I*, 𝜔 = 0.5… 2.5 ppm, and fitted to Eq. (4):

4 $${I}_{{\rm{G}}}={I}_{0}{{\rm{e}}}^{-{G}^{2}{{\rm{\gamma }}}^{2}{{\rm{\delta }}}^{2}D(\Delta -\frac{{\rm{\delta }}}{3})},$$

where γ is the gyromagnetic ratio, δ is the gradient length, Δ is the diffusion time and *D* is the calculated diffusion coefficient^69^.

### NMR Titration of UBA2 from Rad23 to Ub_2_

Unlabeled UBA2 from Rad23 has been stepwise added to Lys11-, Lys27-, and Lys63-linked Ub_2_s in which the proximal domain has been ^15^N isotopically labeled. A series of two-dimensional heteronuclear ^1^H-^15^N HSQC spectra has been acquired to monitor the structural impact UBA2 has on artificially-linked Ub_2_. The titration of UBA2 to Lys11-linked Ub_2_ has been performed by using starting concentrations of *c*^Ub^ = 130 *μ*M and *c*^UBA2^ = 540 *μ*M, respectively, allowing a final [UBA2]/[Ub_2_] ratio of 3.1. The titration of UBA2 to Lys27-linked Ub_2_ has been performed by using starting concentrations of *c*^Ub^ = 80 *μ*M and *c*^UBA2^ = 600 *μ*M, respectively, allowing a final [UBA2]/[Ub_2_] ratio of 2.4. The titration of UBA2 to Lys63-linked Ub_2_ has been performed by using starting concentrations of *c*^Ub^ = 40 *μ*M and *c*^UBA2^ = 600 *μ*M, respectively, allowing a final [UBA2]/[Ub_2_] ratio of 4.6. Changes in chemical shifts have been computed according to Eq. (1). The dissociation constant, *K*_D_, characterizing the interaction between UBA2 and Ub_2_ has been determined by

5 $${\Delta {\rm{\omega }}}^{{\rm{obs}}}={\Delta {\rm{\omega }}}^{{\rm{\max }}}\frac{n{[P]}_{{\rm{t}}}+{[L]}_{{\rm{t}}}+{K}_{{\rm{D}}}-\sqrt{{(n{[P]}_{{\rm{t}}}+{[L]}_{{\rm{t}}}+{K}_{{\rm{D}}})}^{2}-4n{[P]}_{{\rm{t}}}{[L]}_{{\rm{t}}}}}{2n{[P]}_{{\rm{t}}}},$$

where Δω^obs^ represents the change in chemical shift per point of titration, Δω^max^ the maximum of the change in chemical shift, *n* the stoichiometry of binding, [*P*]_t_ the entire concentration of Ub_2_ and [*L*]_t_ the entire UBA2 concentration.

### Molecular dynamics simulations

The following type of MD simulations have been used to aid the interpretation of the NMR data: atomistic simulations of wild type Ub monomers as well as individually cysteine mutated Ub monomers at the Lys11, Lys27, and Lys63 positions, respectively (simulation details are given below). In addition, we present simulation data of Lys11-, Lys27-, and Lys63-linked Ub_2_s, which are covalently linked with a native isopeptide bond. As basis for these data, we have relied on an extensive previous study where we have combined coarse grained (CG) and atomistic simulations with mathematical analysis methods to characterize the conformational ensembles of all natively occurring isopeptide-linked Ub_2_s^14^.

Atomistic MD simulations were performed with the GROMACS simulation package v5^70^. Temperature and pressure were kept at *T* = 300 K and *p* = 1 bar using the velocity rescaling thermostat and the Parrinello-Rahman barostat, respectively. The Verlet cut-off scheme was applied. The LINCS algorithm was used to constrain all bonds. The default md (leap-frog) integrator was used with an integration time step of 2 fs. All MD simulations in this study were performed with the GROMOS96 54a7 force field^71^ and the SPC/E water model. A cut-off for short range van der Waals interactions of 1.4 nm was used. Electrostatics were treated with the Particle Mesh Ewald scheme with a 1.4 nm cut-off^72^.

All MD trajectory analyses were performed either with tools which are available inside the GROMACS package or custom python scripts. Solvent accessible surface area (SASA) calculations were performed with a probe radius of 0.14 nm. *RMSF* values were calculated for backbone atoms over time windows of 10 ns after alignment of all structures to an average structure inside regarding time window. To identify representative structures for monomeric Ub a hierarchy based clustering was performed. For each simulation a pair-wise root mean square deviation (RMSD) matrix was calculated (using backbone atom positions of residues 1 to 72) of trajectory snapshots taken every 100 ps. A hierarchical clustering into 12 clusters was performed as it is implemented in the python module *scipy.cluster.hierarchy* using the Ward method^73^. In each case the first three most populated clusters contained at least 85% of the conformations used as input. For each of these clusters a representative centroid structure was determined and used for illustration.

Initial conformations for Ub monomer simulations were generated from the crystal structure of Ub (PDB ID 1UBQ). For simulations of cysteine mutated monomeric Ub, single lysine residues were replaced with cysteine using the PyMOL software. Production simulations for wild type monomeric Ub were carried out for 4000 ns to obtain a reliable reference data set. Each monomeric cysteine mutated Ub was simulated for 2000 ns.

Conformations for atomistic simulations of Ub_2_s were obtained from an ensemble generated by CG simulations and subsequent back-mapping to the atomistic level^14^.

The general workflow used to obtain conformational ensembles for Ub_2_s from atomistic simulations is graphically summarized in Fig. S19. An equilibrated atomistic ensemble of Ub_2_ – which includes transitions between different domain-domain interfaces and states of mutual orientation of the two Ub_2_ moieties with respect to each other – is hardly accessible by standard atomistic MD simulations. We show this exemplarily with the help of a 5000 ns long atomistic simulation of Ub_2_ in Fig. S19A where a domain-domain interface is formed directly after the start and preserved for the rest of the time – with only minor rearrangements of the initially formed Ub_2_ conformation. In contrast, in a CG simulation of Ub_2_ multiple domain-domain interfaces are formed and disbanded during the same simulation time of 5000 ns, but with a computational effort which is smaller by a factor of 200. Thanks to this acceleration we were able to obtain an equilibrated CG ensemble for all possible Ub_2_ linkage types (a total of 120 µs simulation time for each linkage type) which show remarkably good agreement both with experimental results^37,74^ and with atomistic simulations that were carried out for validation^14^. We used a combination of dimensionality reduction and clustering to draw two-dimensional (2D) free energy landscapes of domain-domain orientations and to identify conformational states of Ub_2_, in particular with regards to connecting the different orientations and domain-domain binding interfaces with experimentally found linkage-specific behavior. We used a set of 144 minimum distances (one distance for each residue for Ub_2_ inside of the globular core excluding the flexible C-termini) as a descriptor (collective variables) to characterize each Ub_2_ conformation. This set of collective variables is based on Cα atoms and thus can be applied to compare conformations from simulations of different linkage types and different levels of resolution (atomistic and coarse grained) but also experimental structures. This is achieved by dimensionality reduction of this 144D vector which gives a point in 2D for each Ub_2_ conformation and with this a way for intuitive comparison. This allows also to obtain linkage specific 2D free energy landscapes from which one can extract low free energy structures for back-mapping to the atomistic scale (Fig. S19B). This procedure has been described in full detail previously^14,39^.

Herein, we had already validated the shape of the sampled landscapes by multiple long free atomistic simulations and probed the stability of low-free energy structures by reinserting atomistic details into CG conformations and initiating atomistic simulations of various length^14^. For the present study we have used back-mapped conformations of Ub_2_s (four for each linkage type, representing the deepest free energy minima) and performed 100 ns long atomistic simulations for each conformation (Fig. S19C). After back-mapping, atomistic structures were relaxed by energy minimization before and after solvation. Solvated systems were equilibrated in three short runs of 200 ps: (1) under constant temperature (NVT) with a position restrained backbone; (2) under constant temperature and pressure (NPT) with a position restrained backbone; (3) NPT without any position restraints. During the atomistic simulations we observed no major conformational rearrangements which confirmed our earlier observations that (i) the CG low-free-energy structures are compatible with the atomistic model and (ii) on the atomistic-simulation level transitions between different domain-domain interfaces are extremely elusive. Since the atomistic ensembles presented here are based on the most significant Ub_2_ conformations they have (taken-together) been used as representative for the ensemble of the different Ub_2_ types in solution.

## Supplementary information

Supplementary Information

## Data Availability

The NMR resonance assignments have been deposited in the Biological Magnetic Resonance Data Bank with the following accession numbers: 27803 (UbK11C), 27802 (UbK11C-PA), 27804 (UbK11C-linked dimer), 27806 (UbK27C), 27805 (UbK27C-PA), 27807 (UbK27C-linked dimer), 27809 (UbK63C), 27808 (UbK63C-PA), and 27810 (UbK63C-linked dimer).
