# Supplementary material for: Conformational and functional characterization of artificially conjugated non-canonical ubiquitin dimers
Source: Sci Rep. 2019 Dec 27;9:19991. doi: 10.1038/s41598-019-56458-z (PMC6934565; doi:10.1038/s41598-019-56458-z)
Supplement: Supplementary file 1 — Supplementary Information [file 41598_2019_56458_MOESM1_ESM.pdf]

Supplementary Information

Conformational and functional characterization of artificially conjugated non-canonical ubiquitin dimers

Tobias Schneider[1,3], Andrej Berg[1,3], Zeynel Ulusoy[2,3], Martin Gamerdinger[2,3], Christine Peter[1,3], and Michael Kovermann[1,3,4*]

[1] Department of Chemistry, Universitätsstrasse 10, Universität Konstanz, DE-78457 Konstanz

[2] Department of Biology, Universitätsstrasse 10, Universität Konstanz, DE-78457 Konstanz

[3] Graduate School Chemical Biology KoRS-CB, Universitätsstrasse 10, Universität Konstanz, DE-78457 Konstanz

[4] Zukunftskolleg, Universitätsstrasse 10, Universität Konstanz, DE-78457 Konstanz

correspondence: michael.kovermann@uni-konstanz.de

Content:

Figures S1-S19

Table S1

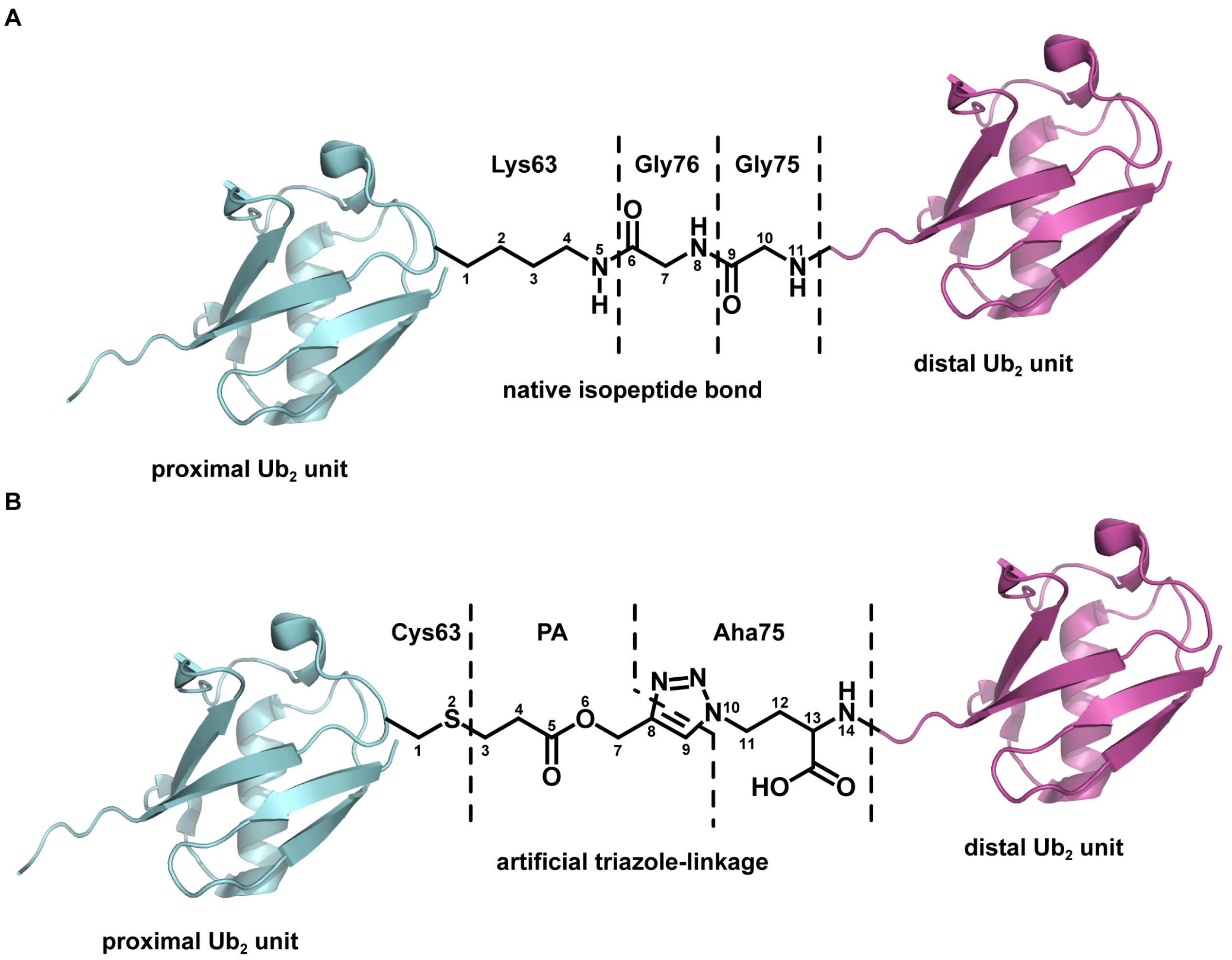

FIGURE S1 Illustration of Lys63-linked Ub_2 comprising either the native isopeptide bond (A) or the artificial triazole-linkage (B) used for the NMR experiments in this study. Note that in the distal unit of the artificially triazole-linked Ub_2 from (B) Gly76 is deleted and Gly75 is replaced by the unnatural amino acid azidohomoalanine (Aha75) to retain, approximately, the same distance between the proximal and the distal moiety as in the natively isopeptide-linked Ub_2 form presented in (A). The structures have been created by using the PyMOL Molecular Graphics System, Version 2.4.0a0, Schrödinger, LCC (www.pymol.org) and ChemDraw 18 (www.perkinelmer.com).

A

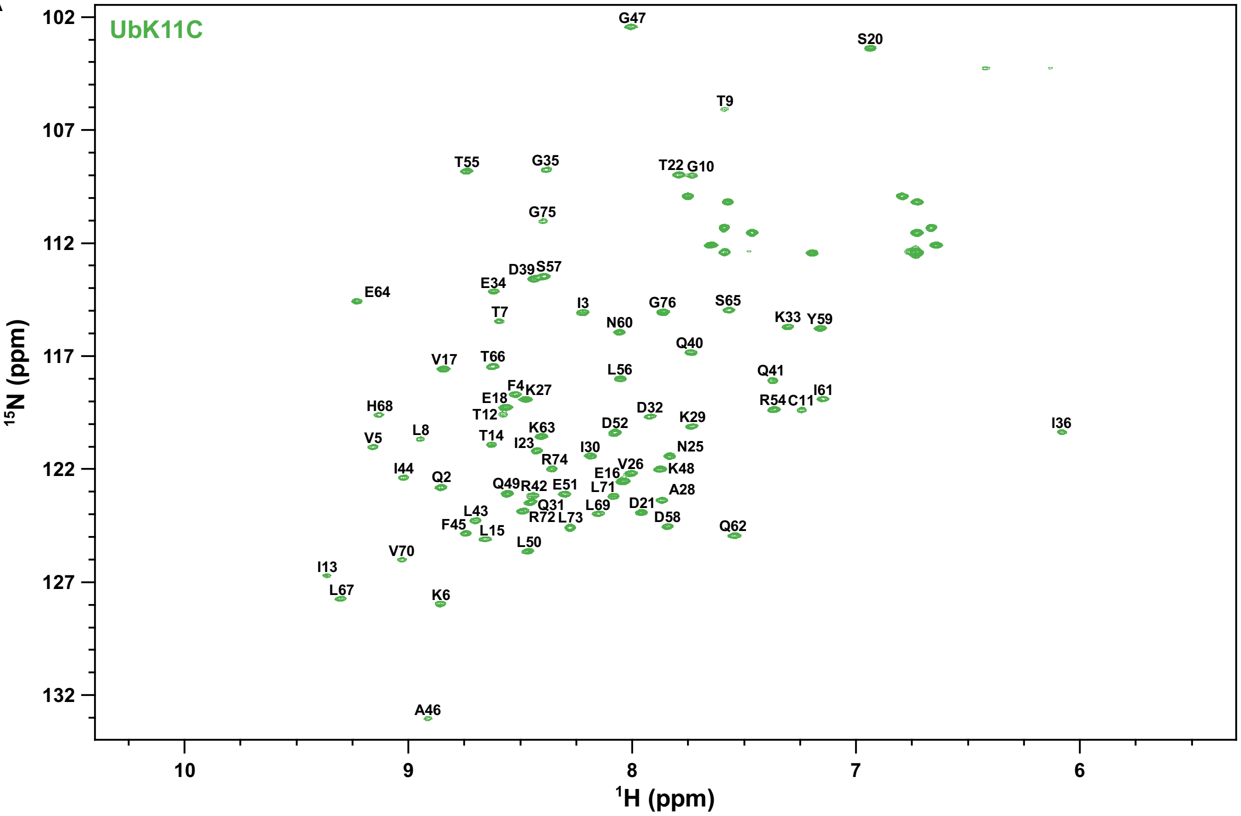
UbK11C
G47
S20
T9
T55
G35
T22
G10
G75
D39
S57
E34
E64
I3
G76
S65
T7
N60
K33
Y59
Q40
V17
T66
L56
Q41
F4
K27
E18
I61
R54
C11
H68
T12
D32
K29
I36
D52
K63
L8
V5
T14
I23
I30
N25
R74
V26
E16
K48
I44
Q2
Q49
R42
E51
L71
A28
Q31
L69
D21
L43
R72
L73
D58
F45
L15
Q62
L50
V70
I13
L67
K6
A46
102
107
112
117
122
127
132
^{15}N (ppm)
10
9
8
7
6
^{1}H (ppm)

B

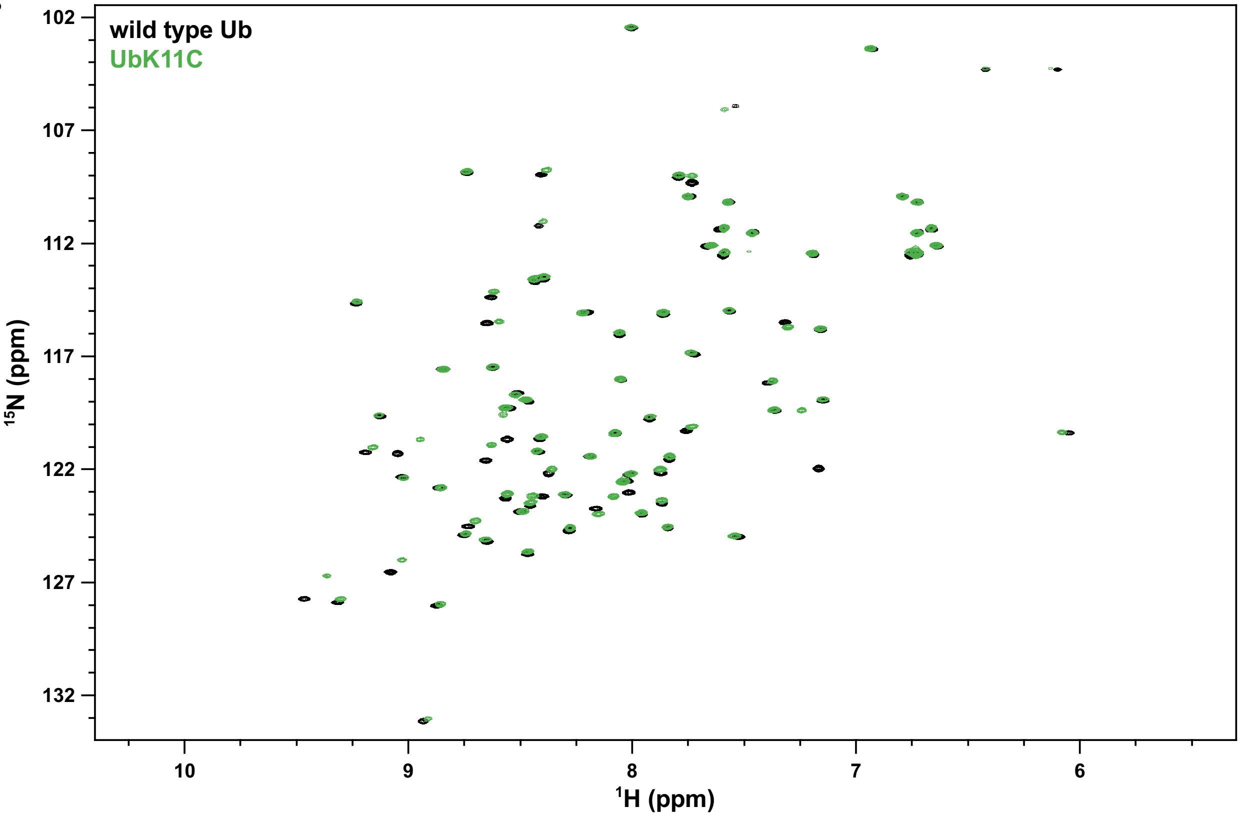
wild type Ub
UbK11C
102
107
112
117
122
127
132
^{15}N (ppm)
10
9
8
7
6
^{1}H (ppm)

C

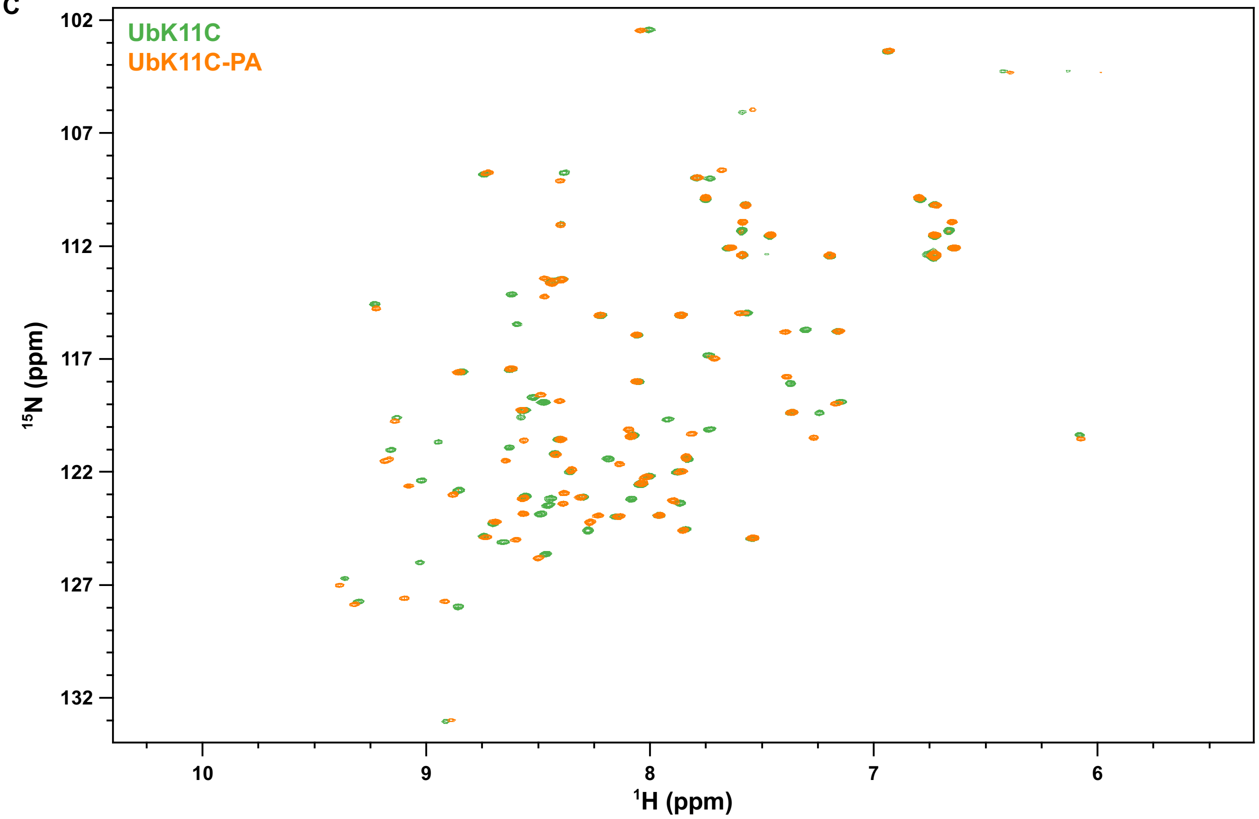

UbK11C
UbK11C-PA
102
107
112
117
122
127
132
^{15}N (ppm)
10
9
8
7
6
^{1}H (ppm)

D

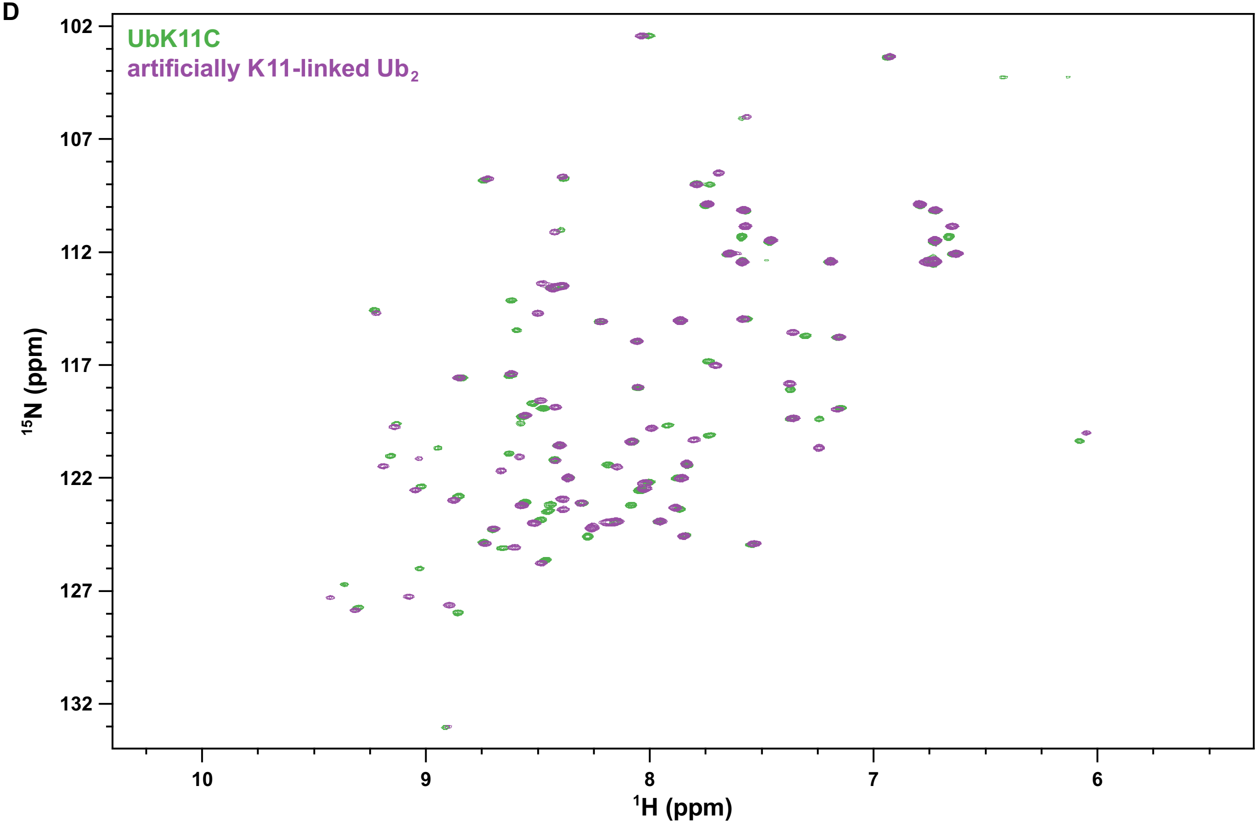

UbK11C
artificially K11-linked Ub$_2$
102
107
112
117
122
127
132
^{15}N (ppm)
10
9
8
7
6
^{1}H (ppm)

FIGURE S2 Two-dimensional heteronuclear ^{1}H-^{15}N HSQC spectra of Ub species involved in the generation of artificially Lys11-linked Ub_2. Assignment of cross signals of monomeric UbK11C is displayed in (A) by using the one letter code for amino acids followed by the position in the primary sequence. The same spectrum is superimposed with monomeric wild type Ub in (B) referring to CSP values displayed in **Fig. 1A**. Spectra of monomeric UbK11C lacking or possessing PA linker are superimposed in (C) referring to CSP values displayed in **Fig. 1B**. Spectra of monomeric UbK11C and the corresponding artificially PA-linked Ub_2 illuminating cross signals comprising the proximal unit are superimposed in (D) referring to CSP values displayed in **Fig. 1C**.

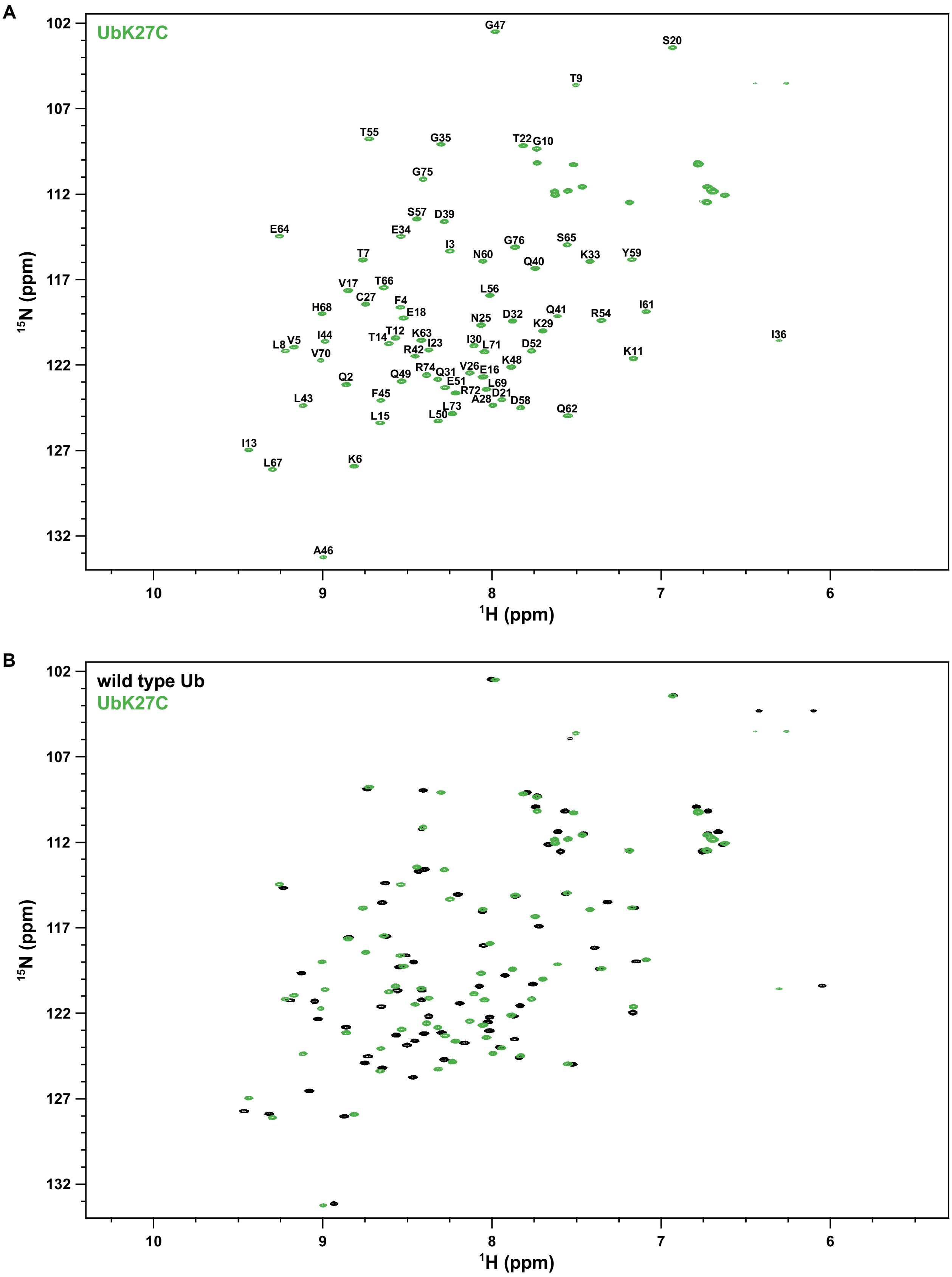
A
UbK27C
G47
S20
T9
T55
G35
T22
G10
G75
S57
D39
E64
E34
I3
G76
S65
T7
N60
K33
Y59
Q40
V17
T66
L56
C27
F4
H68
E18
I61
D32
Q41
R54
N25
K29
I44
T12
K63
I36
T14
I23
I30
L8
V5
L71
D52
R42
V70
K11
K48
R74
V26
E16
Q31
Q49
Q2
E51
L69
R72
F45
D21
L43
A28
D58
L73
Q62
L15
L50
I13
K6
L67
A46
B
wild type Ub
UbK27C
102
107
112
117
122
127
132
10
9
8
7
6
^{15}N (ppm)
^{1}H (ppm)

C

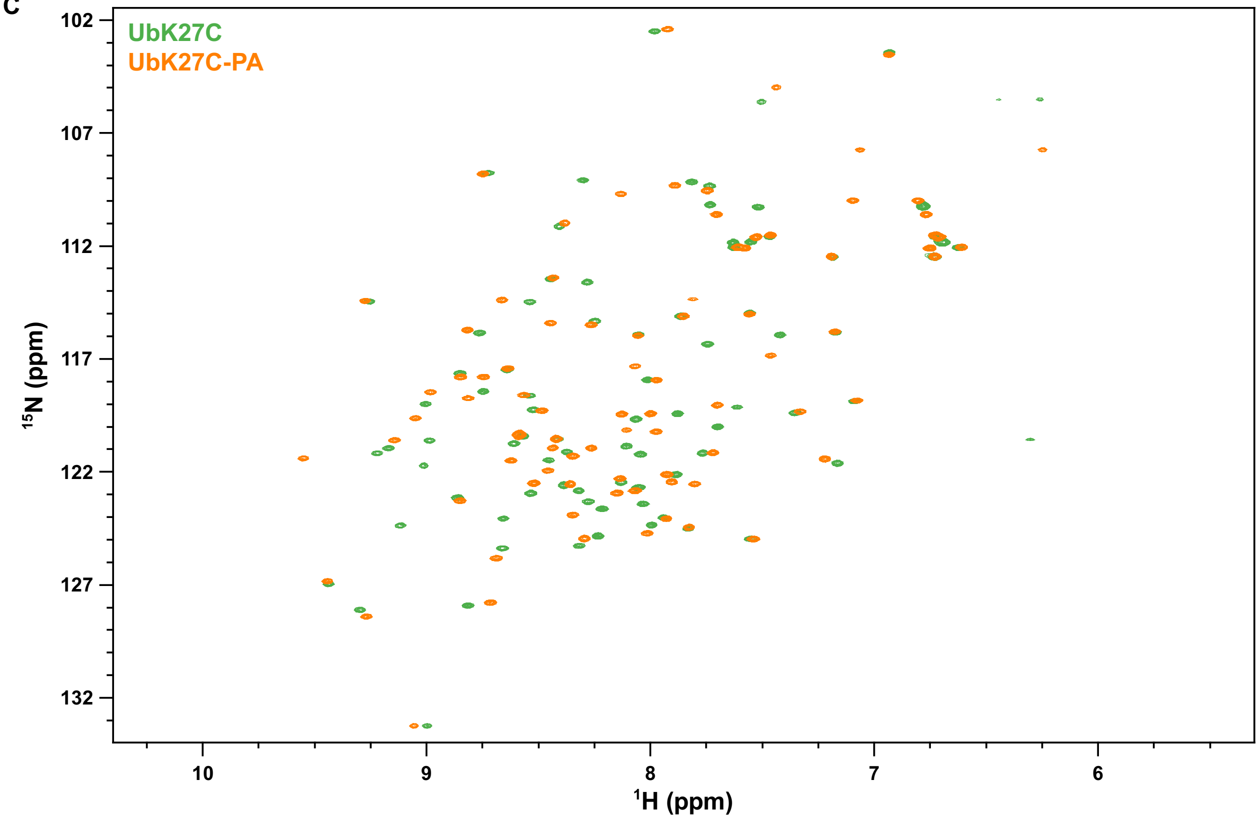
UbK27C
UbK27C-PA
102
107
112
117
122
127
132
^{15}N (ppm)
10
9
8
7
6
^{1}H (ppm)

D

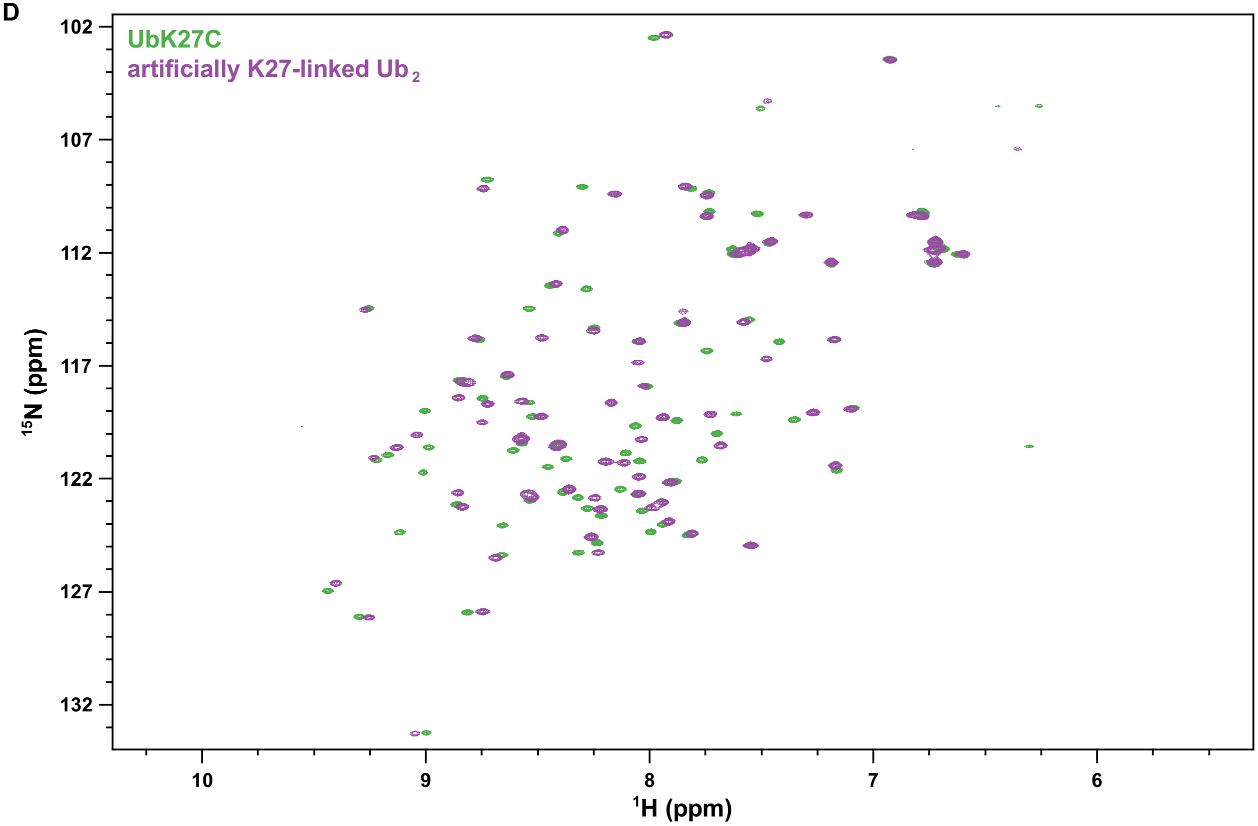
UbK27C
artificially K27-linked Ub$_2$
102
107
112
117
122
127
132
^{15}N (ppm)
10
9
8
7
6
^{1}H (ppm)

FIGURE S3 Two-dimensional heteronuclear ^{1}H-^{15}N HSQC spectra of Ub species involved in the generation of artificially Lys27-linked Ub_2. Assignment of cross signals of monomeric UbK27C is displayed in (A) by using the one letter code for amino acids followed by the position in the primary sequence. The same spectrum is superimposed with monomeric wild type Ub in (B) referring to CSP values displayed in **Fig. 1A**. Spectra of monomeric UbK27C lacking or possessing PA linker are superimposed in (C) referring to CSP values displayed in **Fig. 1B**. Spectra of monomeric UbK27C and the corresponding artificially PA-linked Ub_2 illuminating cross signals comprising the proximal unit are superimposed in (D) referring to CSP values displayed in **Fig. 1C**.

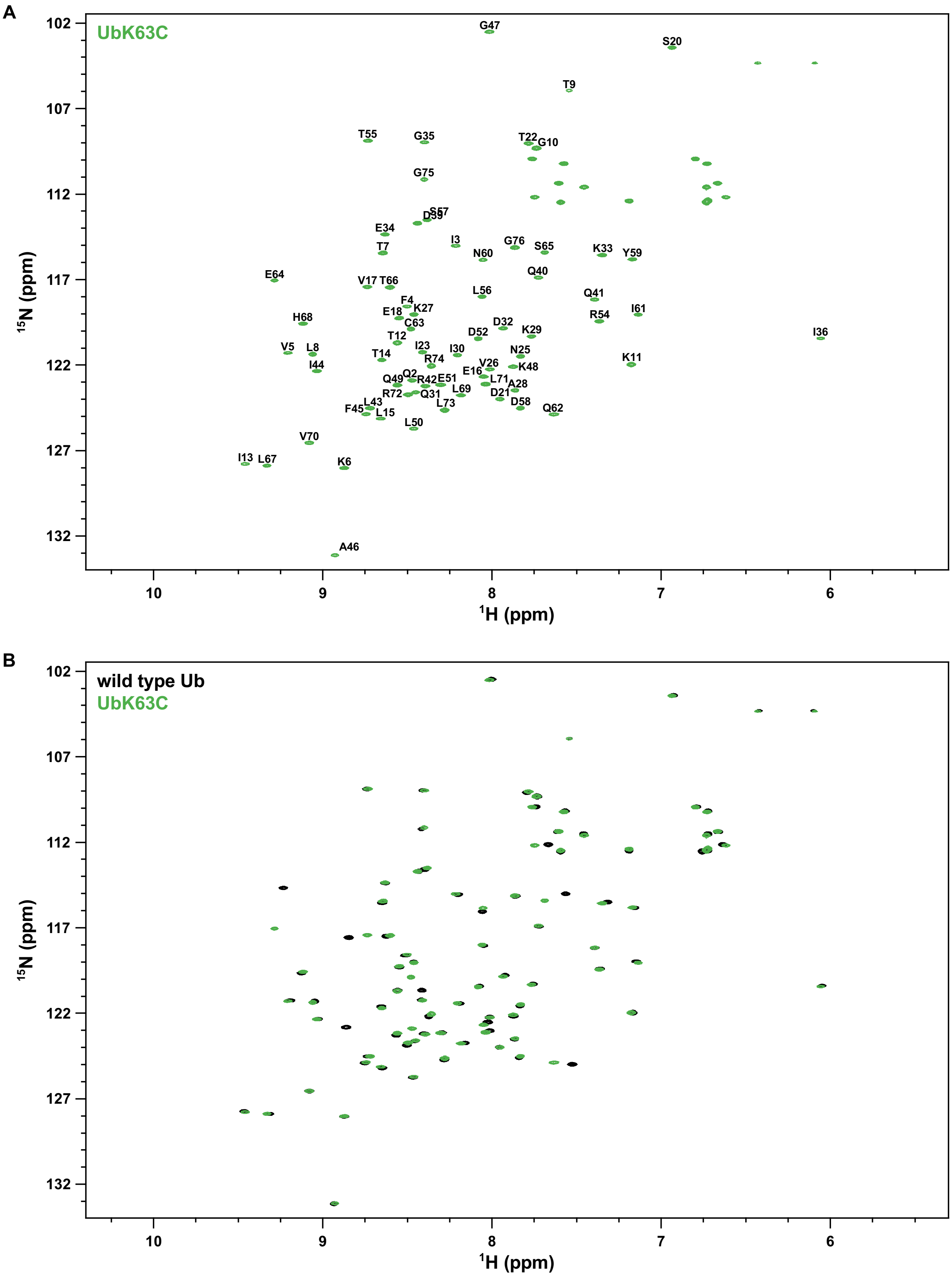
A
UbK63C
G47
S20
T9
T55
G35
T22
G10
G75
S57
D39
E34
T7
I3
G76
S65
N60
K33
Y59
E64
Q40
V17
T66
L56
Q41
F4
K27
E18
H68
C63
R54
I61
D32
T12
D52
K29
I36
V5
L8
T14
I23
I30
R74
N25
K11
I44
V26
E16
K48
Q49
Q2
R42
E51
L71
A28
R72
Q31
L69
D21
L43
L73
D58
F45
L15
Q62
L50
V70
I13
L67
K6
A46
^{15}N (ppm)
^{1}H (ppm)
B
wild type Ub
UbK63C
^{15}N (ppm)
^{1}H (ppm)

C

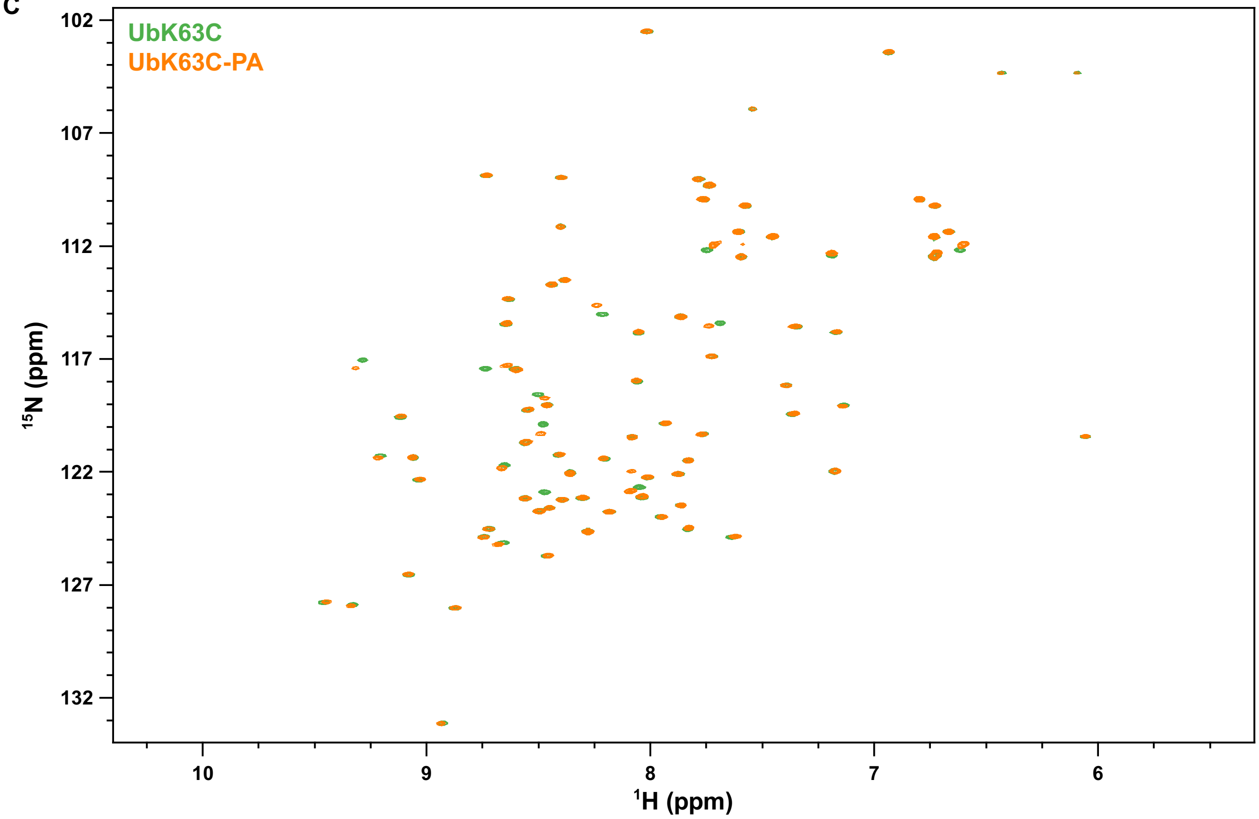
UbK63C
UbK63C-PA
102
107
112
117
122
127
132
^{15}N (ppm)
10
9
8
7
6
^{1}H (ppm)

D

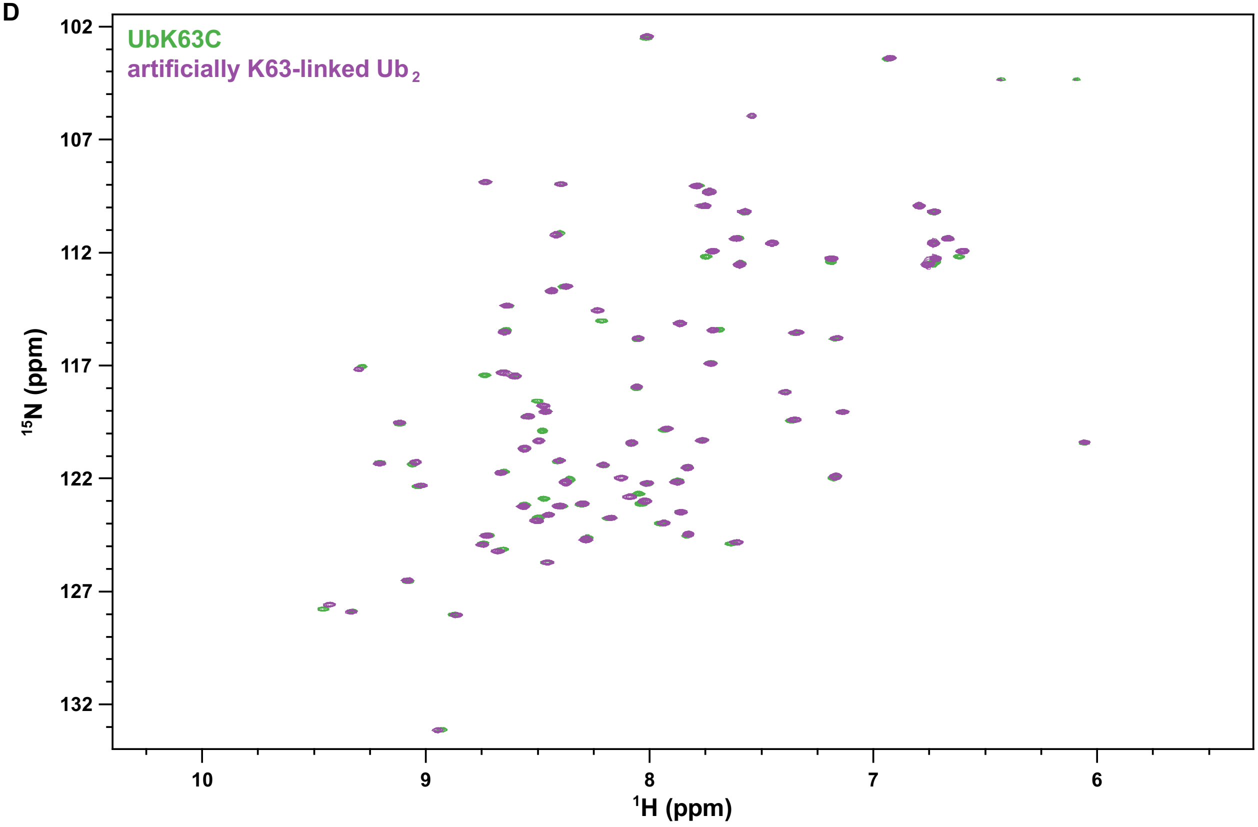
UbK63C
artificially K63-linked Ub_2
102
107
112
117
122
127
132
^{15}N (ppm)
10
9
8
7
6
^{1}H (ppm)

FIGURE S4 Two-dimensional heteronuclear ^{1}H-^{15}N HSQC spectra of Ub species involved in the generation of artificially Lys63-linked Ub_2. Assignment of cross signals of monomeric UbK63C is displayed in (A) by using the one letter code for amino acids followed by the position in the primary sequence. The same spectrum is superimposed with monomeric wild type Ub in (B) referring to CSP values displayed in **Fig. 1A**. Spectra of monomeric UbK63C lacking or possessing PA linker are superimposed in (C) referring to CSP values displayed in **Fig. 1B**. Spectra of monomeric UbK63C and the corresponding artificially PA-linked Ub_2 illuminating cross signals comprising the proximal unit are superimposed in (D) referring to CSP values displayed in **Fig. 1C**.

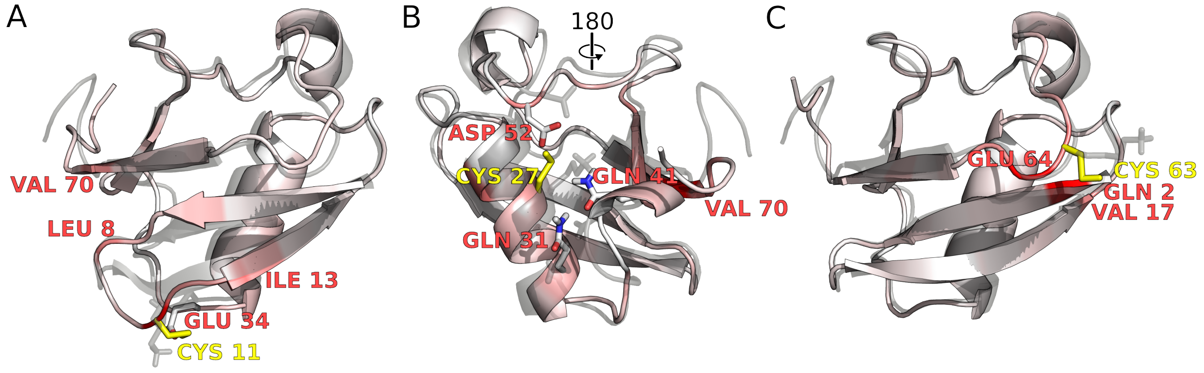

FIGURE S5 Superimposition of protein structures obtained from atomistic MD simulations performed for monomeric wild type Ub (colored in light gray; the lysine residues are shown in stick mode) and monomeric cysteine mutants UbK11C (A), UbK27C (B) and UbK63C (C) (color ranges from gray to red according to the differences in chemical shifts between monomeric wild type Ub and the respective cysteine mutants determined by NMR spectroscopy as presented in **Fig. 1A**). The alignment has been performed by using the Cα atoms comprising Met1 to Arg72. The cysteine residues have been labelled and colored in yellow according to the primary sequence and are shown in stick mode. Residues which are significantly affected by lysine to cysteine mutation as illuminated by CSP analysis (Fig. 1A) have been additionally labeled in red. UbK27C has been rotated by 180 degrees in (B) enabling the view to the mutation site. The structures have been created by using the PyMOL Molecular Graphics System, Version 1.8.4.0, Schrödinger, LCC (www.pymol.org).

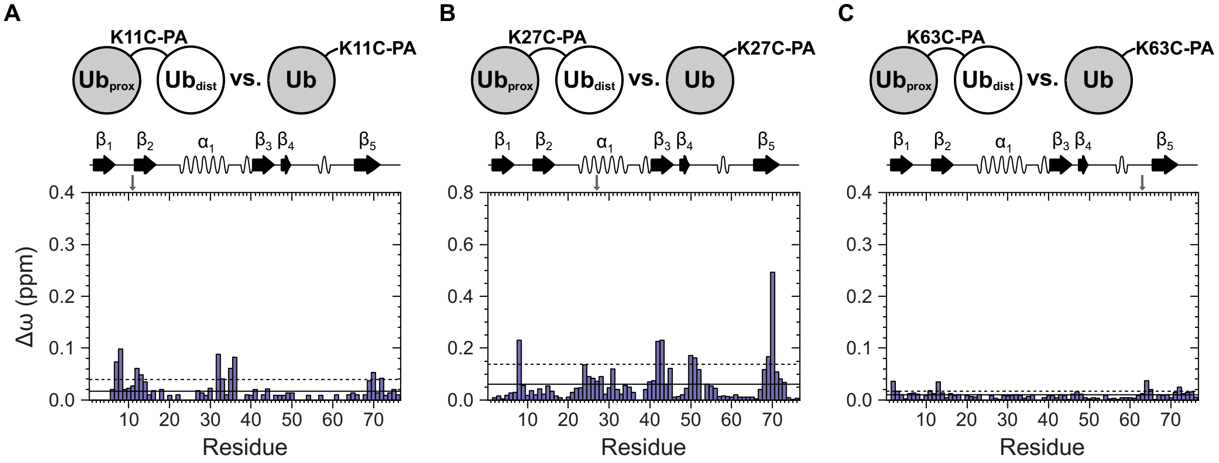

FIGURE S6 Analysis of chemical shift perturbations (Δω) based on the proximal unit of artificially Lys11- (A), Lys27- (B) or Lys63-linked Ub_2 (C) versus corresponding monomeric cysteine mutated Ub possessing the PA linker (note the different scaling in B compared to A and C). The horizontal lines indicate Δω values larger than the mean (continuous mode) as larger than the mean plus one standard deviation (dotted mode). Secondary structural elements according to PDB ID 1D3Z are indicated on top and the site used for conjugation has been highlighted by using a vertical arrow. Note that supplemental Δω values are shown in **Fig. 1**.

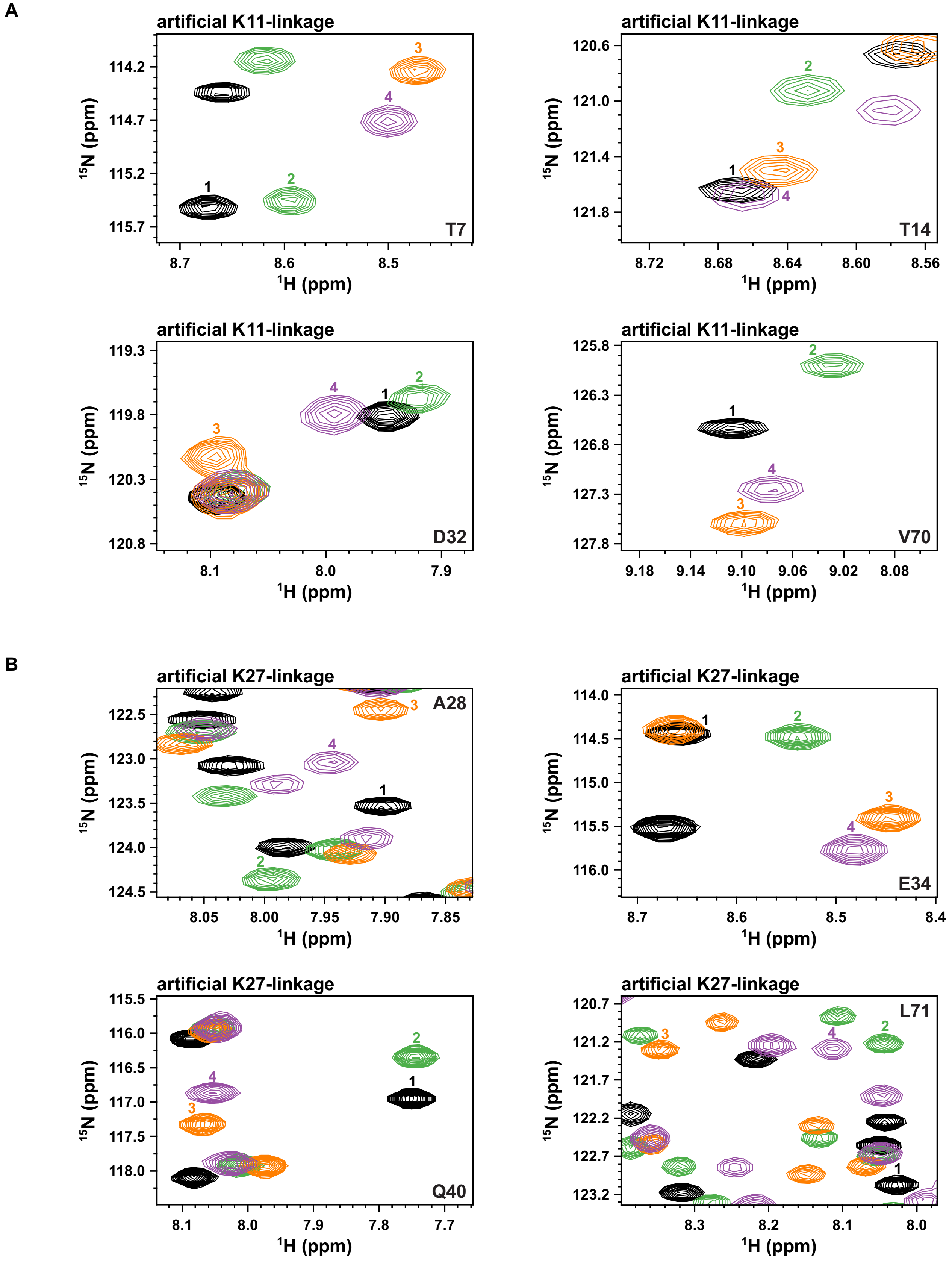
A
artificial K11-linkage
T7
^{15}N (ppm)
114.2
114.7
115.2
115.7
8.7
8.6
8.5
^{1}H (ppm)
T14
120.6
121.0
121.4
121.8
8.72
8.68
8.64
8.60
8.56
D32
119.3
119.8
120.3
120.8
8.1
8.0
7.9
V70
125.8
126.3
126.8
127.3
127.8
9.18
9.14
9.10
9.06
9.02
8.08
B
artificial K27-linkage
A28
122.5
123.0
123.5
124.0
124.5
8.05
8.00
7.95
7.90
7.85
E34
114.0
114.5
115.0
115.5
116.0
8.7
8.6
8.5
8.4
Q40
115.5
116.0
116.5
117.0
117.5
118.0
8.1
8.0
7.9
7.8
7.7
L71
120.7
121.2
121.7
122.2
122.7
123.2
8.3
8.2
8.1
8.0

C

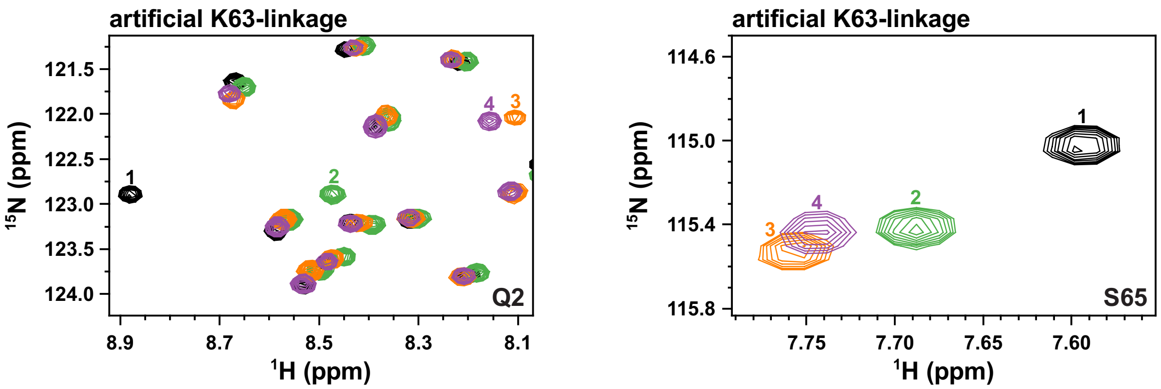

FIGURE S7 Illustration of the change of chemical shifts obtained for different Ub variants by acquiring two-dimensional heteronuclear ^{1}H-^{15}N HSQC NMR spectra. The spectra have been color coded and labeled in the following manner: monomeric wild type Ub is colored in black and labeled by 1, monomeric cysteine mutated Ubs are colored in green and labeled by 2, monomeric cysteine mutated Ubs possessing the PA linker are colored in orange and labeled by 3 and the proximal moieties of artificially linked Ub_2s are colored in purple and labeled by 4, respectively. The labeling of the cross signals indicated on the right (bold) refers to the position in the primary sequence in Ub and by additional use of the one letter code for amino acids. The site used for artificial linkage is indicated on top of the individual two-dimensional ^{1}H-^{15}N HSQC NMR spectra, namely Lys11-linkage in (A), Lys27-linkage in (B), and Lys63-linkage in (C).

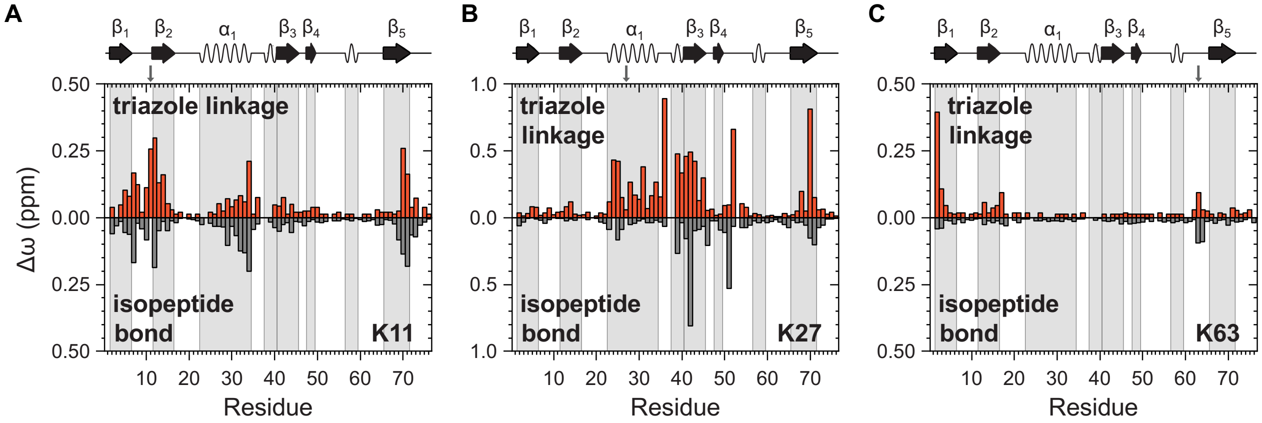

FIGURE S8 Comparison of CSP values for residues comprising the proximal moiety originated from artificial conjugation of Ub_2 based on triazole linkage as discussed in this study with CSP values obtained from isopeptide conjugation. The comparison has been done for Lys11- (A), Lys27- (B), and Lys63-linkage (C). The data shown for isopeptide conjugation have been published by Castañeda et *al.* (PCCP, 2016). Note that the analysis of changes of chemical shifts for all types of linkages has been done by applying an identical equation: $\Delta\omega = ((\Delta\omega^{1H})^2 + (\Delta\omega^{15N}/5)^2)^{0.5}$.

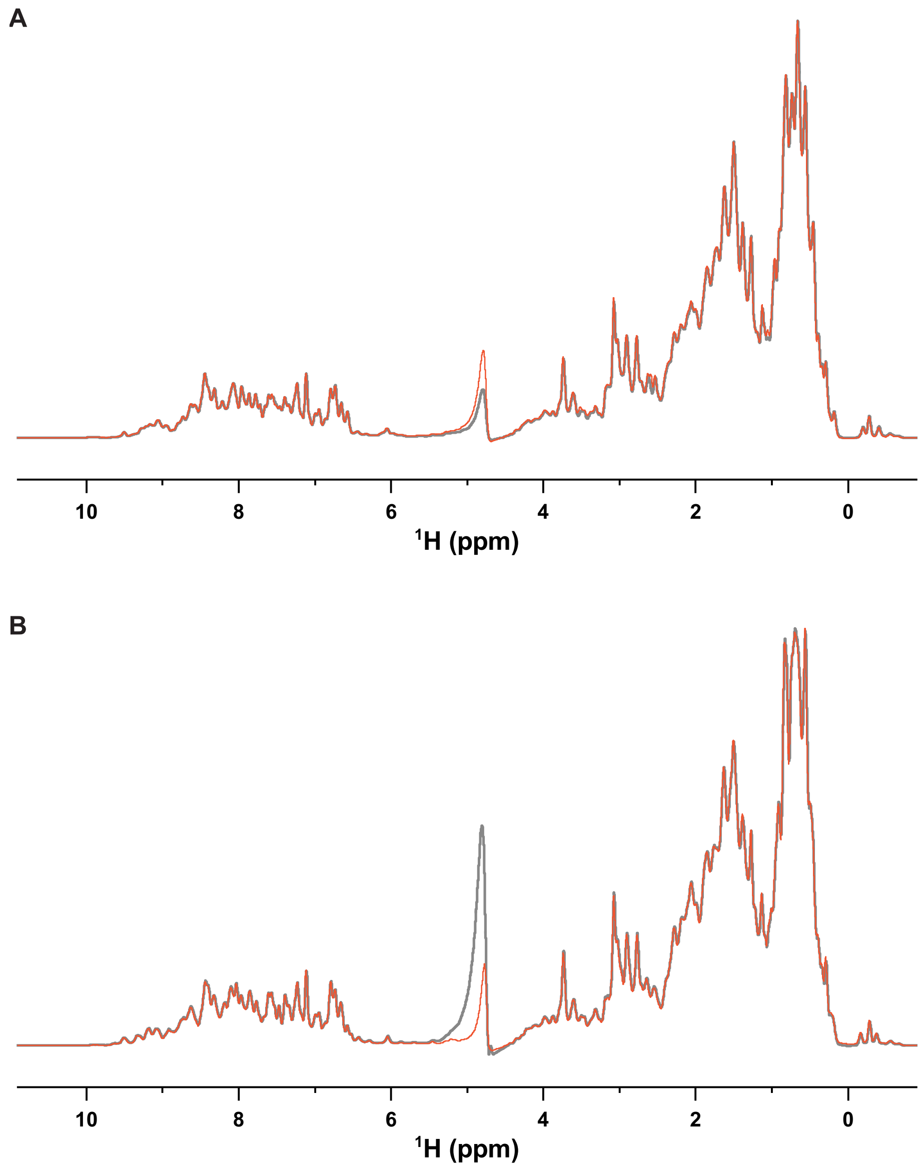

FIGURE S9 One-dimensional ^{1}H NMR spectra of artificially Lys11- (A) and Lys27-linked Ub_2 (B) before (colored in gray) and after (colored in red) long-term storage at T = 253 K demonstrating the strong stability of the triazole linkage used for domain-domain conjugation. A period of 14 (Lys11-linkage) and 13 months (Lys27-linkage) elapsed between measurements. No additional ingredients beside 20 mM Na_3PO_4 and 5 % (*v*/*v*) D_2O have been added to both proteins.

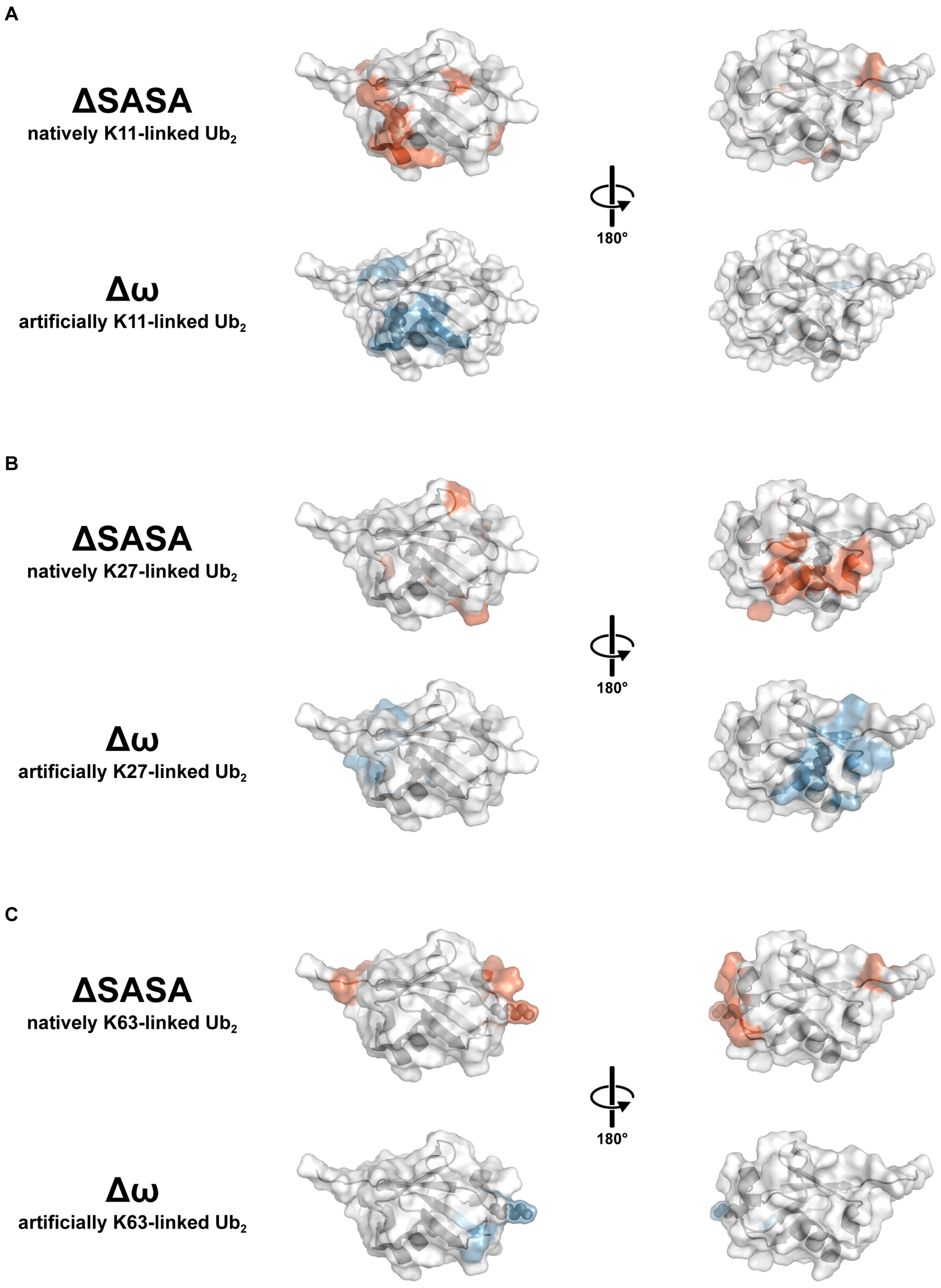
A
ΔSASA
natively K11-linked Ub2
Δω
artificially K11-linked Ub2
180°
B
ΔSASA
natively K27-linked Ub2
Δω
artificially K27-linked Ub2
180°
C
ΔSASA
natively K63-linked Ub2
Δω
artificially K63-linked Ub2
180°

FIGURE S10 Surface representation of monomeric wild type Ub (PDB ID 1D3Z) representing simulated ΔSASA values comparing the proximal units of natively isopeptide-linked Ub_2s with monomeric wild type Ub (colored in orange). Moreover, experimental $\Delta\omega$ values are shown comparing the proximal units of artificially PA-linked Ub_2s with the corresponding monomeric cysteine mutated Ub variants (colored in blue), respectively. Residues comprising the proximal moiety in Lys11- (A), Lys27- (B), and Lys63-linkage type (C) have been highlighted by using red or blue color once they possess ΔSASA or $\Delta\omega$ values larger than the mean (**Fig. 2**). The structures have been created by using the PyMOL Molecular Graphics System, Version 2.4.0a0, Schrödinger, LCC (www.pymol.org).

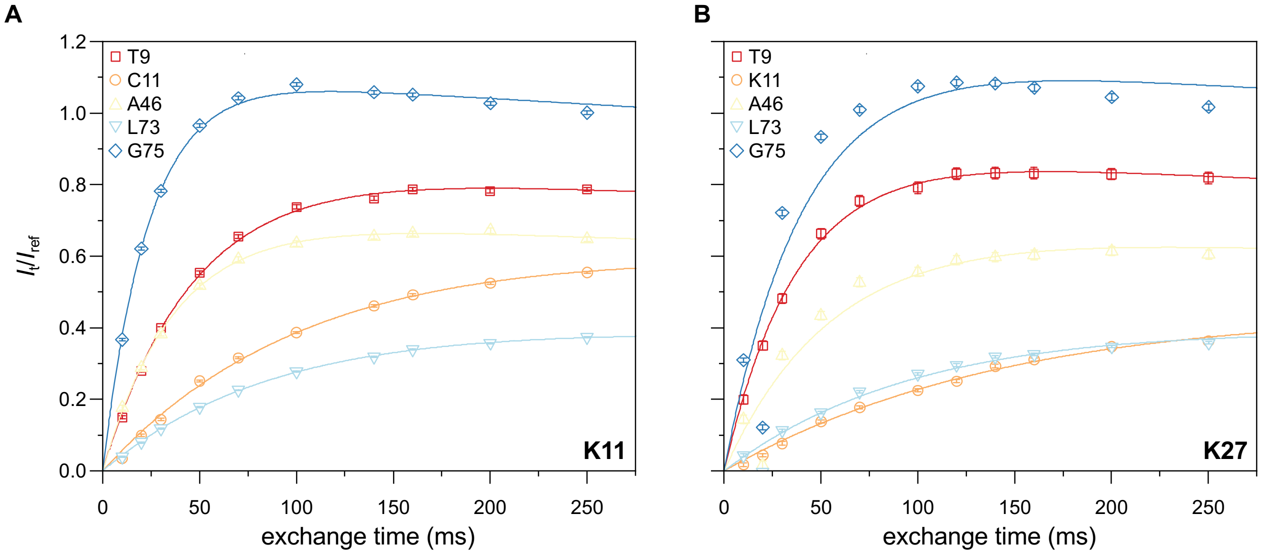

FIGURE S11 Build-up of normalized intensities for selected residues obtained by applying the modified MEXICO NMR experiment. The continuous lines show fitting of equation (2) to the experimental data obtained for selected residues (using one letter code followed by the position in primary sequence) comprising the proximal moiety of artificially Lys11- (A) or Lys27-linked Ub_2 (B). This enables to obtain the rate constant, k_{HX}, characterizing amide proton to solvents proton exchange taking place on a millisecond time scale. The calculated k_{HX} values are presented in **Fig. 3A** and individual k_{HX} values are summarized in **Table S1**.

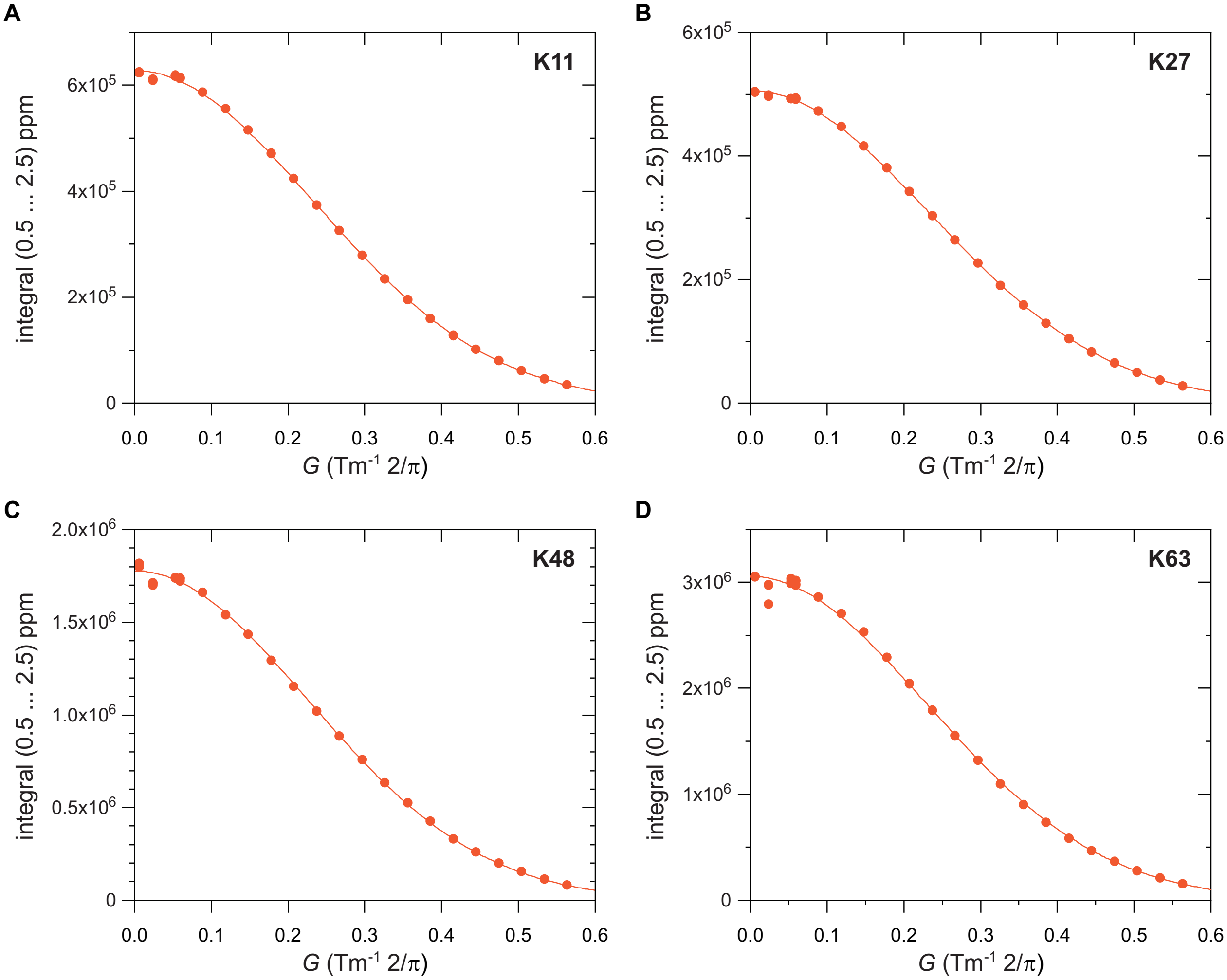

FIGURE S12 One dimensional ^{1}H NMR spectra of Lys11- (A), Lys27- (B), Lys48- (C), and Lys63-linked Ub_2 (D) have been integrated in the aliphatic signal region ranging between 0.5 and 2.5 ppm at varying gradient field strengths. The integrals (shown as filled circles) have been subsequently used for the determination of the diffusion coefficient. The straight lines represent the best fits of Eq. (4) to the experimental data yielding D = (8.95±0.08)*10^{-11} m^2s^{-1} (A), D = (8.89±0.04)*10^{-11} m^2s^{-1} (B), D = (9.5±0.1)*10^{-11} m^2s^{-1} (C), and D = (9.2±0.2)*10^{-11} m^2s^{-1} (D).

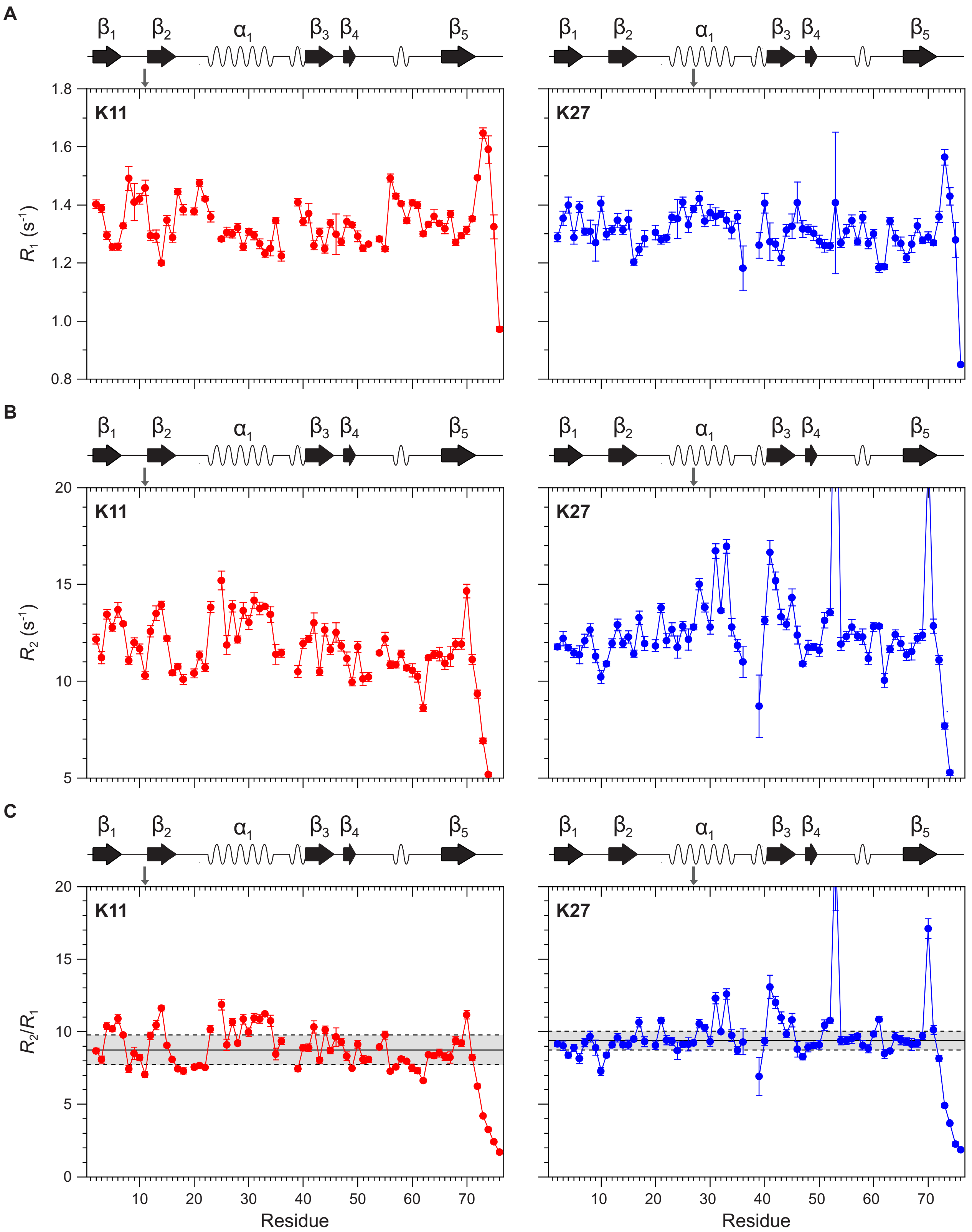
A
B
C
β1 β2 α1 β3 β4 β5
K11
K27
R1 (s-1)
R2 (s-1)
R2/R1
Residue

FIGURE S13 Longitudinal (R_1) (A) and transversal relaxation rate constants (R_2) (B) as well as R_2/R_1 values (C) for individual residues comprising the proximal units of artificially PA-linked Ub_2s originating from Lys11- (colored in red) and Lys27-linkage (colored in blue). Secondary structural elements according to the NMR solution structure (PDB ID 1D3Z) are indicated on top in (A), (B) and (C). The solid line in (C) indicates the 10 % trimmed mean of R_2/R_1 values representing residues comprising the proximal unit of artificially Lys11- and Lys27-linked Ub_2s. The dashed lines mark one standard deviation.

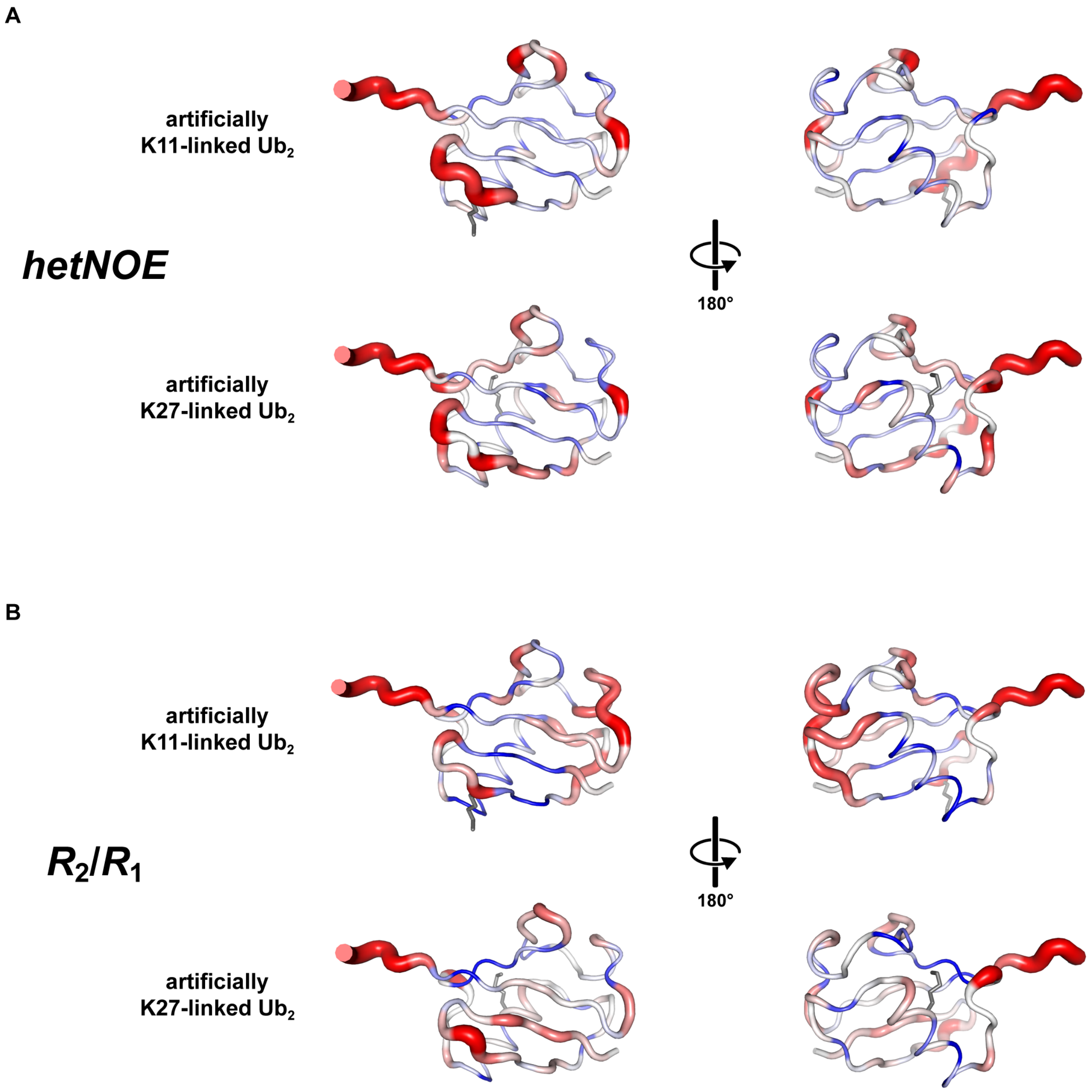

FIGURE S14 NMR relaxation data obtained for the proximal units of artificially Lys11- and Lys27-linked Ub_2s are highlighted on the structure of monomeric wild type Ub (PDB ID 1D3Z). *HetNOE* values (A) shown in **Fig. 4A** and R_2/R_1 values (B) shown in **Fig. 13C** are represented using putty mode. Low *hetNOE* values and R_2/R_1 values below the 10 % trimmed mean indicating high amplitude motions and fast dynamics on the picosecond to nanosecond time scale, respectively, are highlighted by thick tubes and are colored in red, whereas high *hetNOE* values and R_2/R_1 values above the 10 % trimmed mean indicating low amplitude motions and slow dynamics, respectively, are highlighted by thin tubes and are colored in blue. The side chain of the corresponding lysine residue used for cysteine mutation and

linkage is depicted in sticks mode. The structures have been created by using the PyMOL Molecular Graphics System, Version 2.4.0a0, Schrödinger, LCC (www.pymol.org).

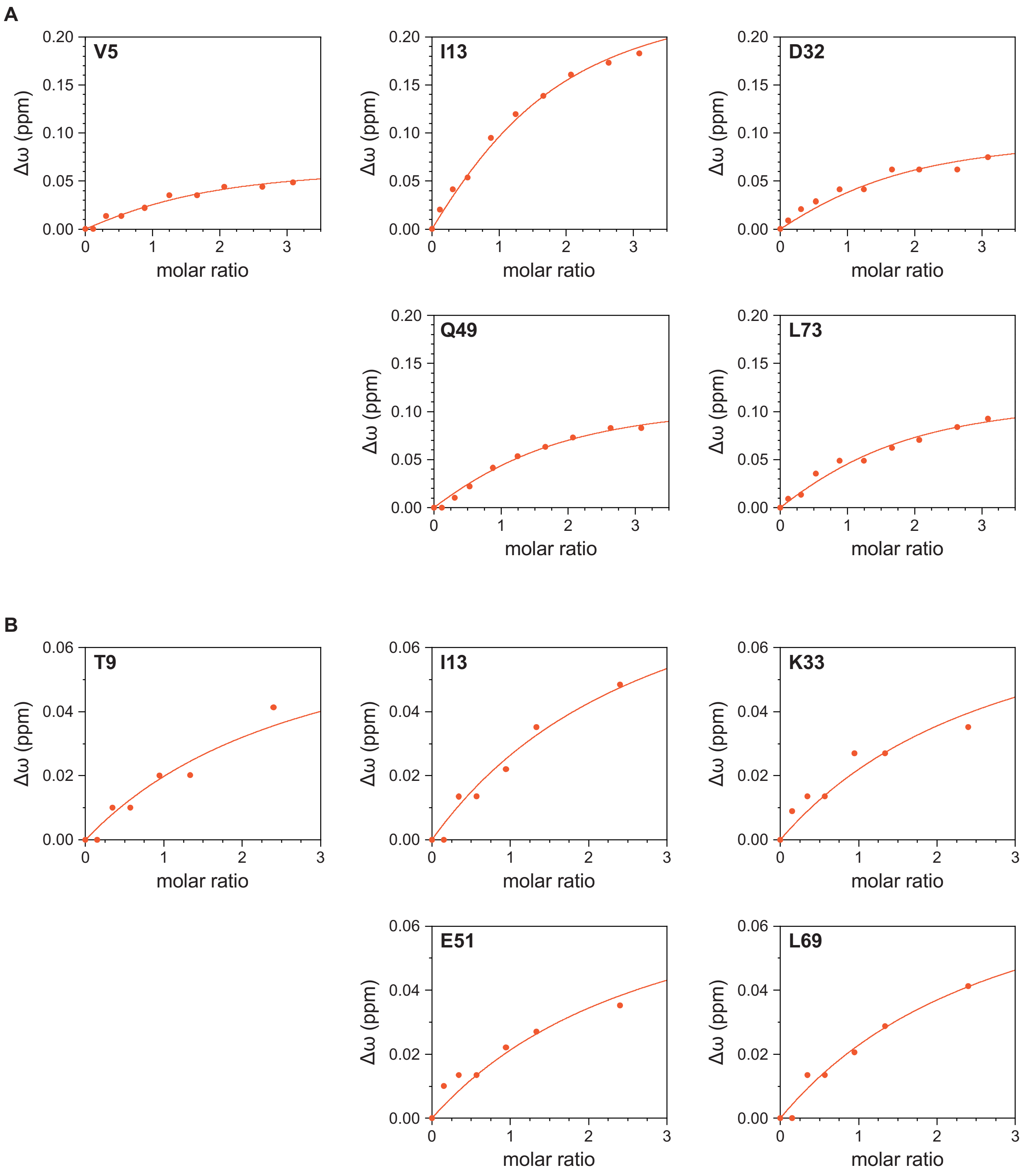
A
V5
I13
D32
Q49
L73
B
T9
I13
K33
E51
L69
Δω (ppm)
molar ratio
0.00
0.05
0.10
0.15
0.20
0.02
0.04
0.06
0
1
2
3

C

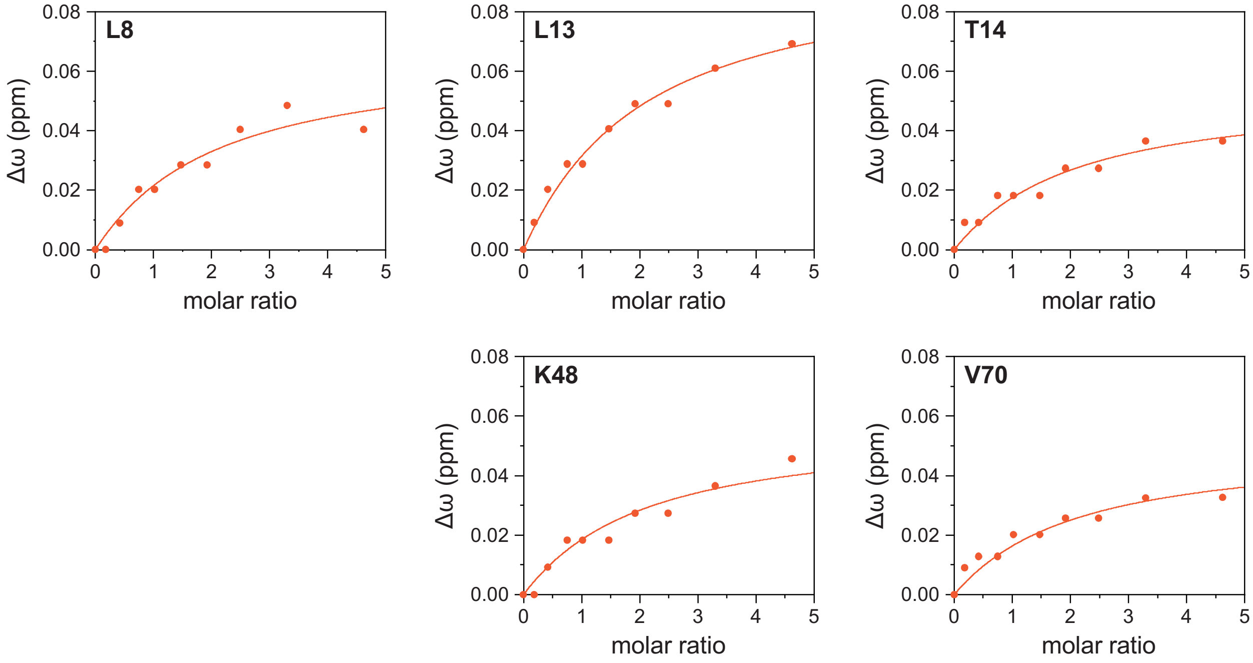

FIGURE S15 Individual NMR titration profiles obtained for adding Rad23 UBA2 to artificially Lys11- (A), Lys27- (B) and Lys63-linked Ub_2s (C) resulting from a global fitting procedure for the dissociation constant, K_D. Representative profiles are depicted for selected residues reaching a significant CSP value at the endpoint of the titration experiment according to Figs. 6C and S16, respectively. A 2:1 binding stoichiometry between Ub_2 and UBA2 has been assumed for Lys11-linkage (A), a 1:1 stoichiometry for Lys27-linkage (B) and a 1:2 binding stoichiometry for Lys63-linkage (C), respectively.

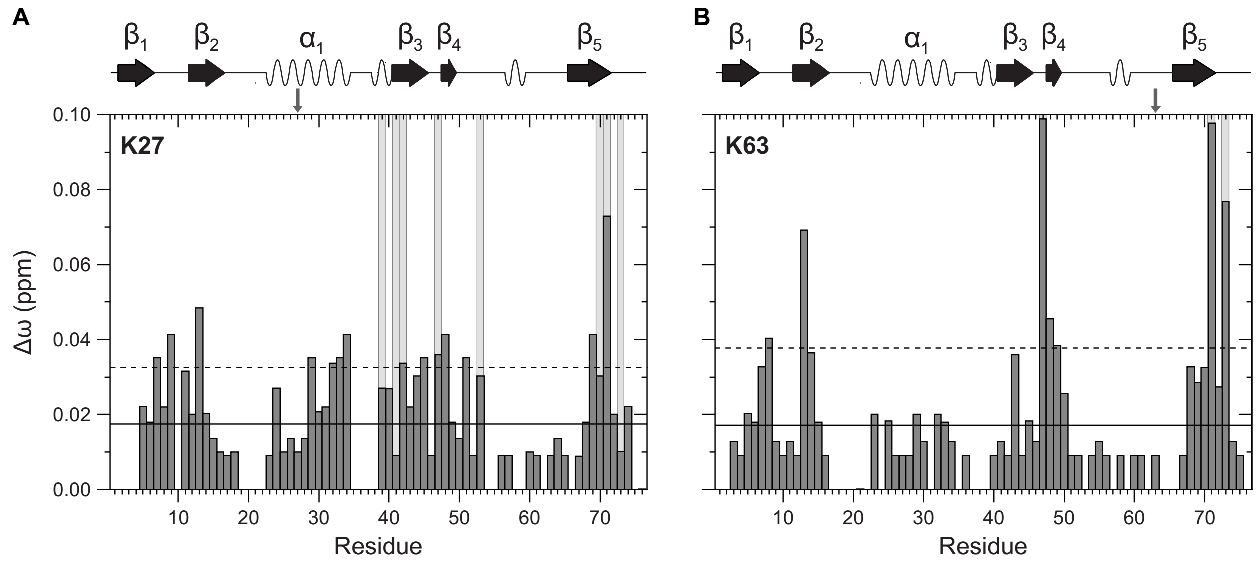

FIGURE S16 Overall change in chemical shifts of residues comprising the proximal moiety in artifcially Lys27- and Lys63-linked Ub_2s comparing absence with presence of a 2.4 (A) or 4.6 times stoichiometric excess (B) of UBA2 regarding Ub_2. The horizontal lines indicate Δω values larger than the mean (continuous mode) and larger than the mean plus one standard deviation (dotted mode). Residues undergoing a signal attenuation larger than 75 % are highlighted by using a background colored in gray. Secondary structural elements according to PDB ID 1D3Z are indicated on top and the site used for conjugation has been highlighted by using a vertical arrow.

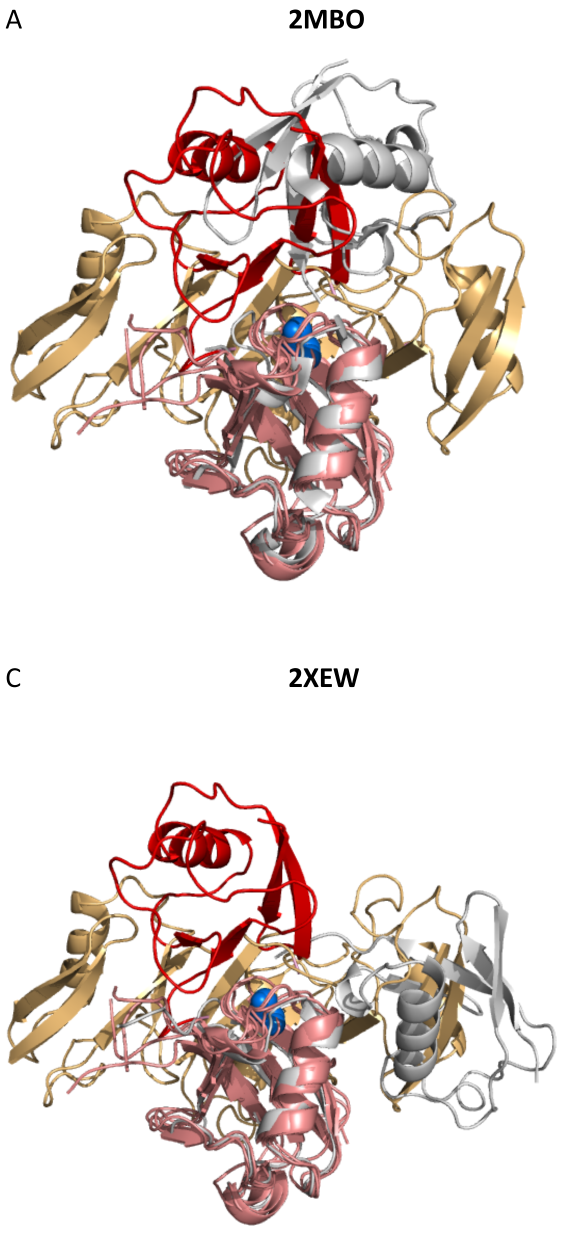

FIGURE S17 Structural alignment performed for Lys11-linked Ub_2s. Firstly, the alignment has been performed for the four structures lowest in free energy obtained in the present study for Lys11-linked Ub_2 (see **Fig. 5, left**) by taking all backbone atoms of residues comprising the proximal moieties into account (A-D, colored in pink). The distal moieties of Lys11-linked Ub_2s have been colored in red (for the structure lowest in free energy) and in orange (for the three remaining ones), respectively (A-D). Secondly, the proximal moieties corresponding to PDB ID 2MBO (A), 2MBQ (B), 2XEW (C), and 3NOB(D) have been individually aligned to the bundle of proximal moieties obtained first and have been colored

in light gray. The distal moieties of 2MBO, 2MBQ, 2XEW, and 3NOB have been colored in light gray, too. Additionally, residue Lys11 addressed for linkage is colored in blue. The structures have been created by using the PyMOL Molecular Graphics System, Version 2.4.0a0, Schrödinger, LCC (www.pymol.org).

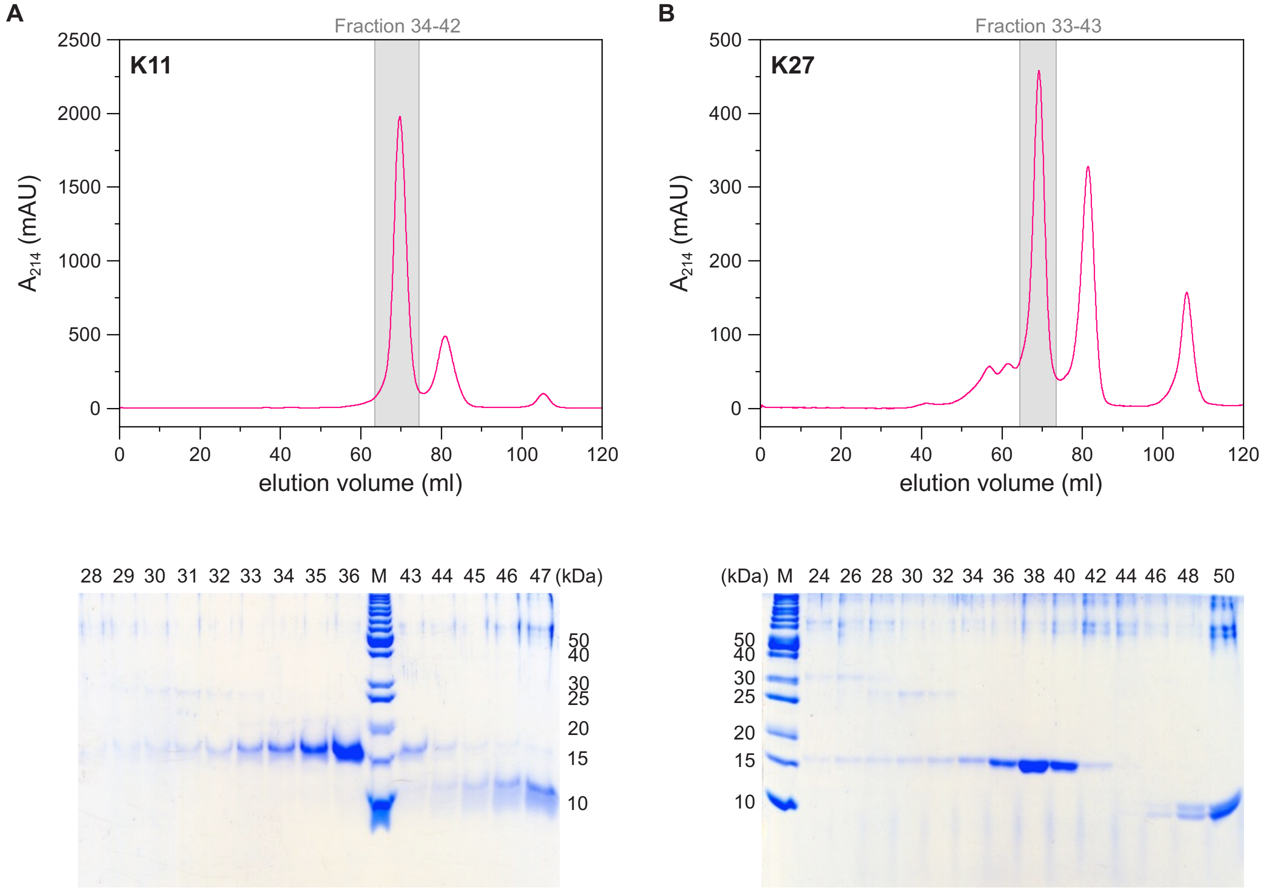

FIGURE S18 Chromatograms obtained for size exclusion chromatography (SEC) applied on artificially Lys11-linked Ub_2 (A, top) and on artificially Lys27-linked Ub_2 (B, top), respectively. Corresponding SDS-PAGE analyses are shown below. SDS-pages indicate the individual fractions obtained from SEC runs (top) and the molecular weight per kDa of the marker which has been used (numbers on the right and on the left, respectively).

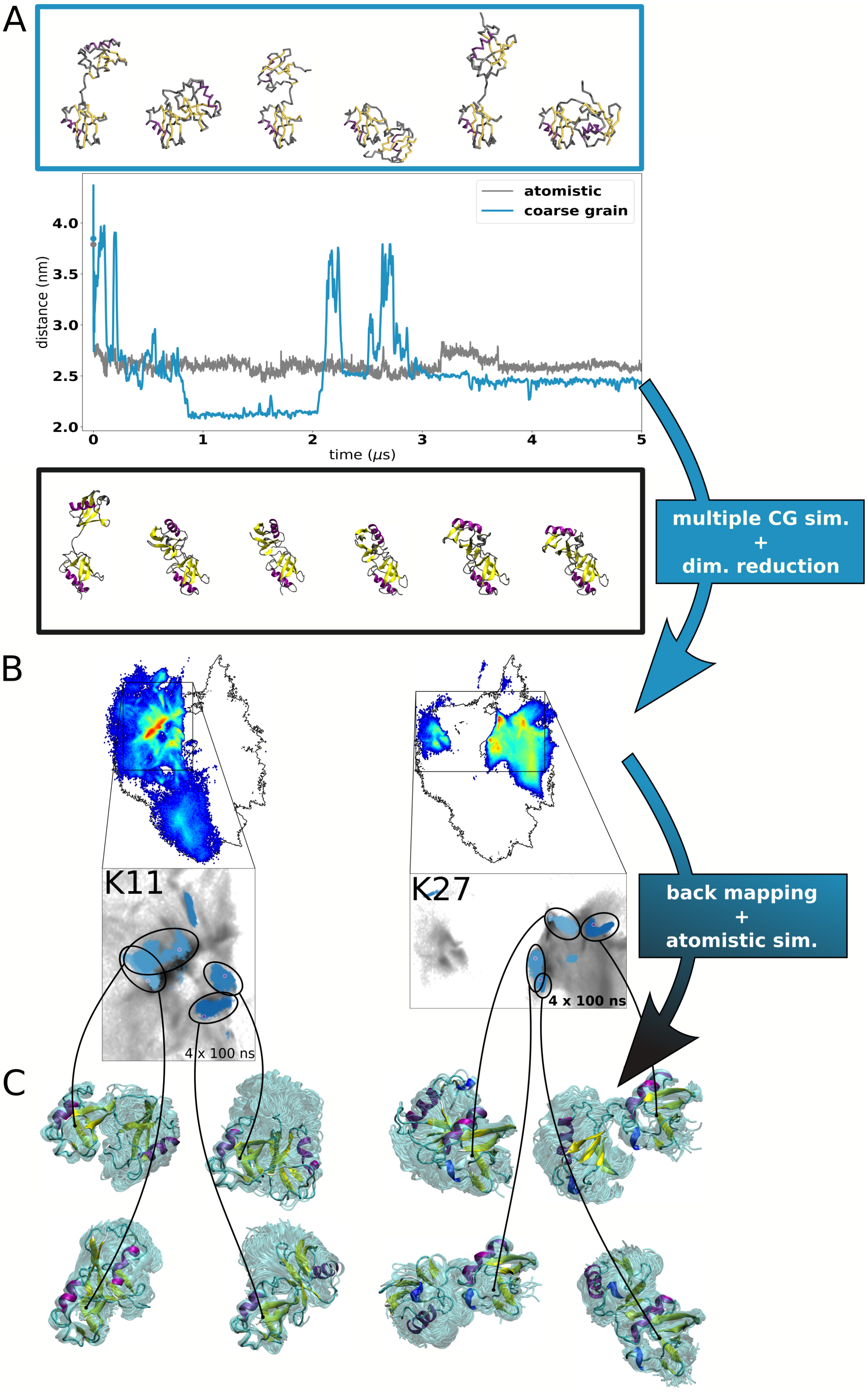
A
atomistic
coarse grain
distance (nm)
time (μs)
multiple CG sim.
+
dim. reduction
B
K11
4 x 100 ns
K27
4 x 100 ns
back mapping
+
atomistic sim.
C

FIGURE S19 Visualization of the general workflow used in the present study to obtain conformational ensembles of Ub_2s on an atomistic scale. (A) Illustration showing that atomistic simulations are not capable to yield converged ensembles of Ub_2. Distance in center of geometry between Ub moieties comprising Ub_2 obtained from atomistic (black) and coarse grained (blue) simulations. Both simulations were started from a fully open conformation. Colored boxes show snapshots of corresponding simulations in cartoon representation (black box, atomistic) or stick representation of backbone beads (blue box, coarse grain). Shortly after initialisation of the atomistic simulation a domain-domain interface is formed and preserved until the end of simulation. In contrast, on coarse grained level Ub_2 takes multiple different conformations. (B) Energy landscapes (colormap) obtained from multiple independent coarse grained simulations of Ub_2 (12 runs x 10 ms for each linkage type) by dimensionality reduction of a set of 144 distances between to two moieties comprising Ub_2. For each type of linkage four coarse grained conformations were extracted from minima possessing the lowest energy and back-mapped to the atomistic scale. Zoom insets show the landscape (grey scale) and conformations from atomistic simulations (blue dots). (C) Structural bundles of Ub_2 obtained from atomistic simulations. The proximal moieties have been aligned and are marked by a line indicating the position of corresponding conformations on low dimensional projection shown in (B). The structures have been created by using the PyMOL Molecular Graphics System, Version 1.8.4.0, Schrödinger, LCC (www.pymol.org).

TABLE S1 Apparent rate constants obtained for amide protons of monomeric wild type Ub and residues comprising the proximal moiety in Lys11-, and Lys27-linked Ub_2 by applying the modified MEXICO approach.

Residue	k_{HX} (s^{-1}) monomeric wild type Ub	k_{HX} (s^{-1}) artificially Lys11-linked Ub_2	k_{HX} (s^{-1}) artificially Lys27-linked Ub_2	Δk_{HX} (s^{-1}) artificially Lys11-linked Ub_2 vs. monomeric wild type Ub	Δk_{HX} (s^{-1}) artificially Lys27-linked Ub_2 vs. monomeric wild type Ub
Gln2	0.61 ±0.01	1.00 ±0.04	0.93 ±0.05	0.40 ±0.04	0.32 ±0.05
Ile3	0.36 ±0.02	0.41 ±0.04	0.42 ±0.10	0.06 ±0.04	0.06 ±0.10
Phe4	0.25 ±0.02	0.27 ±0.02	0.32 ±0.06	0.02 ±0.03	0.07 ±0.07
Val5	0.49 ±0.05	0.34 ±0.04	0.35 ±0.06	-0.15 ±0.07	-0.14 ±0.08
Lys6	1.43 ±0.04	0.64 ±0.07	0.69 ±0.11	-0.79 ±0.08	-0.74 ±0.11
Thr7	0.69 ±0.03	0.95 ±0.04	0.84 ±0.05	0.26 ±0.05	0.15 ±0.06
Leu8	7.84 ±0.06	7.69 ±0.14	3.29 ±0.10	-0.15 ±0.15	-4.55 ±0.11
Thr9	20.84 ±0.48	10.02 ±0.19	12.82 ±0.27	-10.82 ±0.52	-8.02 ±0.55
Gly10	5.07 ±0.08	5.20 ±0.07	2.82 ±0.10	0.12 ±0.11	-2.25 ±0.13
Lys/Cys11	3.07 ±0.08	3.85 ±0.15	2.27 ±0.11	0.78 ±0.17	-0.80 ±0.14
Thr12	6.18 ±0.04	5.65 ±0.08	3.83 ±0.04	-0.53 ±0.09	-2.35 ±0.05
Ile13	0.48 ±0.01	0.58 ±0.06	0.75 ±0.09	0.11 ±0.06	0.27 ±0.09
Thr14	0.94 ±0.02	1.26 ±0.05	3.83 ±0.04	0.32 ±0.06	2.89 ±0.04
Leu15	0.75 ±0.03	0.79 ±0.10	0.81 ±0.12	0.04 ±0.11	0.05 ±0.12
Glu16	1.08 ±0.06	0.74 ±0.04	0.84 ±0.09	-0.34 ±0.07	-0.25 ±0.11
Val17	1.67 ±0.02	1.63 ±0.03	1.79 ±0.19	-0.03 ±0.04	0.13 ±0.20
Glu18	0.51 ±0.02	0.93 ±0.03	0.66 ±0.08	0.42 ±0.03	0.15 ±0.08
Ser20	0.60 ±0.03	0.92 ±0.04	0.91 ±0.06	0.32 ±0.05	0.30 ±0.07
Asp21	0.27 ±0.00	0.38 ±0.02	0.42 ±0.02	0.11 ±0.02	0.14 ±0.02
Thr22	0.77 ±0.03	0.88 ±0.02	1.23 ±0.13	0.11 ±0.04	0.46 ±0.13
Ile23	1.32 ±0.03	0.58 ±0.03	0.91 ±0.07	-0.74 ±0.05	-0.41 ±0.08
Glu24	-	-	0.98 ±0.19	-	-
Asn25	0.68 ±0.02	0.88 ±0.02	1.06 ±0.09	0.20 ±0.03	0.37 ±0.09
Val26	0.18 ±0.01	0.26 ±0.12	0.48 ±0.17	0.09 ±0.12	0.30 ±0.17
Lys/Cys27	0.16 ±0.01	0.44 ±1.37	0.19 ±0.05	0.28 ±1.37	0.03 ±0.05
Ala28	0.08 ±0.00	0.14 ±0.01	0.23 ±0.06	0.07 ±0.01	0.15 ±0.06
Lys29	0.14 ±0.01	0.26 ±0.04	0.20 ±0.03	0.12 ±0.04	0.06 ±0.04
Ile30	0.10 ±0.01	0.12 ±0.03	0.21 ±0.07	0.02 ±0.03	0.11 ±0.07
Gln31	0.09 ±0.01	0.36 ±0.05	0.39 ±0.04	0.26 ±0.05	0.29 ±0.04
Asp32	0.16 ±0.01	0.38 ±0.07	0.54 ±0.05	0.23 ±0.07	0.38 ±0.05
Lys33	0.24 ±0.02	0.40 ±0.08	1.62 ±0.16	0.16 ±0.08	1.38 ±0.16
Glu34	0.10 ±0.01	0.41 ±0.04	0.52 ±0.09	0.31 ±0.04	0.42 ±0.10

Residue	k_{HX} (s^{-1}) monomeric wild type Ub	k_{HX} (s^{-1}) artificially Lys11-linked Ub_2	k_{HX} (s^{-1}) artificially Lys27-linked Ub_2	Δk_{HX} (s^{-1}) artificially Lys11-linked Ub_2 vs. monomeric wild type Ub	Δk_{HX} (s^{-1}) artificially Lys27-linked Ub_2 vs. monomeric wild type Ub
Gly35	0.07 ±0.00	0.27 ±0.04	0.57 ±0.06	0.20 ±0.04	0.51 ±0.06
Ile36	0.41 ±0.11	0.20 ±0.05	-0.55 ±0.70	-0.21 ±0.12	-0.96 ±0.71
Asp39	1.14 ±0.01	0.91 ±0.01	0.44 ±0.04	-0.23 ±0.01	-0.70 ±0.04
Gln40	0.21 ±0.00	0.27 ±0.02	0.87 ±0.14	0.06 ±0.02	0.66 ±0.14
Gln41	0.25 ±0.01	0.38 ±0.02	0.47 ±0.14	0.13 ±0.02	0.22 ±0.14
Arg42	0.30 ±0.01	0.49 ±0.03	0.43 ±0.07	0.20 ±0.03	0.13 ±0.07
Leu43	0.65 ±0.02	0.70 ±0.02	1.00 ±0.11	0.06 ±0.03	0.36 ±0.11
Ile44	0.34 ±0.04	0.41 ±0.10	1.82 ±2.69	0.07 ±0.11	1.47 ±2.69
Phe45	0.81 ±0.02	0.56 ±0.06	0.51 ±0.14	-0.25 ±0.07	-0.29 ±0.15
Ala46	12.25 ±0.09	11.43 ±0.18	7.44 ±1.36	-0.82 ±0.20	-4.82 ±1.36
Gly47	1.59 ±0.03	1.39 ±0.03	1.77 ±0.45	-0.20 ±0.04	0.17 ±0.45
Lys48	0.41 ±1.28	0.24 ±0.01	0.36 ±0.08	-0.17 ±1.28	-0.06 ±1.28
Gln49	1.53 ±0.02	1.53 ±0.02	1.13 ±0.10	-0.01 ±0.03	-0.40 ±0.10
Leu50	0.38 ±0.02	0.43 ±0.04	0.78 ±0.22	0.05 ±0.04	0.40 ±0.22
Glu51	1.13 ±0.01	1.76 ±0.02	1.78 ±0.20	0.64 ±0.02	0.65 ±0.20
Asp52	0.34 ±0.01	0.50 ±0.03	0.65 ±0.08	0.16 ±0.03	0.31 ±0.08
Gly53	-	-	0.78 ±0.54	-	-
Arg54	0.32 ±0.02	0.35 ±0.01	0.45 ±0.03	0.03 ±0.02	0.14 ±0.03
Thr55	0.73 ±0.02	0.72 ±0.03	0.87 ±0.11	-0.01 ±0.03	0.13 ±0.11
Leu56	0.47 ±0.01	0.42 ±0.02	0.44 ±0.04	-0.06 ±0.03	-0.04 ±0.05
Ser57	0.47 ±0.00	0.65 ±0.02	0.71 ±0.04	0.18 ±0.02	0.24 ±0.04
Asp58	0.63 ±0.01	0.79 ±0.03	1.03 ±0.06	0.16 ±0.03	0.41 ±0.06
Tyr59	0.22 ±0.01	0.28 ±0.01	0.47 ±0.84	0.06 ±0.02	0.25 ±0.84
Asn60	0.45 ±0.02	0.38 ±0.02	0.62 ±0.08	-0.07 ±0.03	0.17 ±0.08
Ile61	0.37 ±6.67	0.37 ±5.38	0.41 ±4.74	0.01 ±8.57	0.05 ±8.18
Gln62	0.48 ±0.01	0.63 ±0.03	0.56 ±0.03	0.15 ±0.03	0.08 ±0.04
Lys63	0.91 ±0.02	0.78 ±0.02	0.77 ±0.08	-0.13 ±0.03	-0.14 ±0.08
Glu64	0.22 ±0.01	0.31 ±0.03	0.35 ±0.08	0.10 ±0.04	0.13 ±0.08
Ser65	0.67 ±0.01	0.89 ±0.02	0.89 ±0.06	0.23 ±0.02	0.22 ±0.06
Thr66	0.75 ±0.01	1.30 ±0.03	1.24 ±0.13	0.55 ±0.03	0.49 ±0.13
Leu67	0.70 ±0.03	0.66 ±0.08	0.70 ±0.18	-0.03 ±0.09	0.00 ±0.18
His68	0.36 ±0.02	0.41 ±0.04	2.37 ±2.24	0.06 ±0.05	2.01 ±2.24

Residue	k_{HX} (s^{-1}) monomeric wild type Ub	k_{HX} (s^{-1}) artificially Lys11-linked Ub_2	k_{HX} (s^{-1}) artificially Lys27-linked Ub_2	Δk_{HX} (s^{-1}) artificially Lys11-linked Ub_2 vs. monomeric wild type Ub	Δk_{HX} (s^{-1}) artificially Lys27-linked Ub_2 vs. monomeric wild type Ub
Leu69	0.50 ±0.02	0.48 ±0.05	0.38 ±0.03	-0.02 ±0.05	-0.12 ±0.04
Val70	0.32 ±0.02	0.38 ±0.08	0.80 ±0.07	0.06 ±0.08	0.48 ±0.07
Leu71	0.25 ±0.02	0.44 ±0.03	0.64 ±0.12	0.19 ±0.03	0.40 ±0.12
Arg72	1.23 ±0.01	1.02 ±0.02	0.64 ±0.06	-0.21 ±0.03	-0.59 ±0.06
Leu73	4.32 ±0.04	3.44 ±0.04	3.07 ±0.39	-0.88 ±0.06	-1.25 ±0.40
Arg74	12.09 ±0.13	9.30 ±0.10	7.28 ±1.28	-2.79 ±0.17	-4.81 ±1.29
Gly75	25.78 ±0.46	22.26 ±0.44	13.57 ±2.74	-3.52 ±0.64	-12.22 ±2.78
Gly76	1.33 ±0.01	1.19 ±0.02	1.25 ±0.24	-0.13 ±0.02	-0.08 ±0.24
